# War or Peace? How the Subjective Perception of Great Power Interdependence Shapes Preemptive Defensive Aggression

**DOI:** 10.3389/fpsyg.2017.00864

**Published:** 2017-06-02

**Authors:** Yiming Jing, Peter H. Gries, Yang Li, Adam W. Stivers, Nobuhiro Mifune, D. M. Kuhlman, Liying Bai

**Affiliations:** ^1^Institute for US-China Issues, University of Oklahoma, NormanOK, United States; ^2^Brain Science Institute, Tamagawa UniversityMachida, Japan; ^3^Psychology Department, Gonzaga University, SpokaneWA, United States; ^4^School of Economics and Management, Kochi University of TechnologyKochi, Japan; ^5^Department of Psychological and Brain Sciences, University of Delaware, NewarkDE, United States; ^6^Department of Applied Psychology, Fuzhou UniversityFuzhou, China

**Keywords:** great power conflict, social interdependence, preemptive strikes, international relations, political psychology

## Abstract

Why do great powers with benign intentions end up fighting each other in wars they do not seek? We utilize an incentivized, two-person “Preemptive Strike Game” (PSG) to explore how the subjective perception of great power interdependence shapes defensive aggression against persons from rival great powers. In Study 1, college students from the United States (*N* = 115), China (*N* = 106), and Japan (*N* = 99) made PSG decisions facing each other. This natural experiment revealed that Chinese and Japanese participants (a) made more preemptive attacks against each other and Americans than against their compatriots, and that (b) greater preexisting perceptions of bilateral competition increased intergroup attack rates. In Study 2, adult Americans (*N* = 127) watched real CNN expert interviews portraying United States–China economic interdependence as more positive or negative. This randomized experiment revealed that the more positive portrayal reduced preemptive American strikes against Chinese (but not Japanese), while the more negative portrayal amplified American anger about China’s rise, increasing preemptive attacks against Chinese. We also found, however, that preemptive strikes were primarily defensive and not offensive. Interventions to reduce defensive aggression and promote great power peace are discussed.

## Introduction

“*The greater the threat, the greater is the risk of inaction – and the more compelling the case for taking anticipatory action to defend ourselves…*”

*The National Security Strategy of the United States* (National Security Council, 2006).

The specter of great power conflict hovers over the 21st Century. The rise of China as a regional and global power has unsettled other great powers. In East Asia, Japanese Prime Minister Shinzo Abe has argued that Sino-Japanese relations today parallel Imperial Germany’s early 20th Century challenge to Great Britain, which precipitated World War I (Perlezjan, 2014). United States President Donald Trump and his foreign and economic policy team (e.g., Navarro, 2015) appear to maintain similarly sinister views of China’s rise. American scholars, meanwhile, fret that a possible United States–China power transition increases the risk of another United States–China war (Allison, 2015). For their part, influential Chinese pundits (e.g., Yan, 2015) trumpet a zero-sum view of great power relations today.

Spread through the mass media, such pessimistic depictions of great power relations today create a psychological context that undermines international cooperation. Specifically, when people are led to believe that great power relations are inherently competitive, they are more likely to expect people from rival countries to be hostile, and take preemptive action to defend themselves.

This psychology of preemptive strikes is consequential. There is little reason to believe that elite foreign policy makers are immune to it. Political elites, furthermore, must be responsive to the psychological states of their national publics to maintain the legitimacy of their governments. And elites are not the only people who can kindle conflict. Great power war can be sparked by small-scale conflicts initiated by a local military commander’s overreaction to perceived threat. Regrettably, however, there does not appear to be much research examining the psychological drivers of preemptive violence in the context of real-world international affairs. In the current study, we investigate this issue by conducting micro-level behavioral experiments.

## Security Dilemma, Preemptive Strikes, and Great Power Conflict

Great power relations are central to the study of international relations (IR). Regrettably, mainstream IR theories largely dismiss the possibility of great power cooperation. For power transition theorists (e.g., Organksi and Kugler, 1980), both Imperial Germany and China today are best understood as “revisionist” rising powers on a warpath. They argue that in a Hobbesian, dog-eat-dog world, great power competition leads inevitably to conflict. For “security dilemma” theorists (e.g., Jervis, 1978), great power relations are not necessarily zero-sum, but are nonetheless fraught with danger. States may act to defensively “balance” against perceived threats by building their militaries or making alliances. But other states will tend to fear such policies, leading them to take similar “defensive” measures. This can lead to arms races, spirals of insecurity, and “tragic” wars like World War I that no one wanted.

Supporting the idea of the security dilemma, recent evolutionary and psychological research strongly suggests that humans are motivated to defend themselves against attack (Rusch, 2013; De Dreu et al., 2016). Such defensive aggression can be anticipatory (Yamagishi and Mifune, 2016). For instance, in our interpersonal relationships, we sometimes strike preemptively to eliminate the risk of being harmed (Simunovic et al., 2013; Halevy, 2017). For instance, a person may break up with a partner they prefer to stay with purely out of fear that their partner will break up with them first. In such a situation, there are no winners.

Preemptive strikes are also a defensive tactic in intergroup relations (Böhm et al., 2016), and specifically IR. “If we wait for threats to fully materialize, we will have waited too long,” Bush (2002) warned United States Army cadets in 2002. Americans must “be ready for preemptive action when necessary to defend our liberty and to defend our lives.” The Bush Doctrine of preemptive strikes contributed to the United States decision to invade Iraq in 2003 to eliminate the perceived threat of Saddam Hussein’s weapons of mass destruction (WMD; Record, 2003), which were never proven to have existed. Similarly, defensive preemptive aggression is a likely cause of future United States–China, China-Japan, and other great power conflict in the 21st century.

Will China’s rise and/or a security dilemma precipitate great power conflict in the 21st century? With their structural and distal focus, power transition and security dilemma theories in IR tell us little about the micro-level psychological mechanisms of defensive fear that may act as the proximate cause of great power wars. And while experimental psychological research on intergroup aggression is better equipped to make causal arguments about the drivers of individual behavior, it has largely relied upon artificial groups (i.e., minimal groups; Tajfel, 1970), seldom examining real-world conflicts. This project seeks to fill this research gap, exploring the psychological drivers of the defensive aggression that can lead to great power war in the 21st Century.

## Perceived Outcome Interdependence and Defensive Preemptive Aggression

To explore the psychological drivers of preemptive strikes between persons from rival countries, we build on social interdependence theory (e.g., Deutsch, 1985; for a review, see Johnson, 2003). It maintains that how people perceive socially interdependent situations shapes their choices to cooperate or compete (Halevy et al., 2012a). When people in an interdependent relationship believe that their interests and goals are aligned (positive outcome interdependence), they may have more benign expectations of each other and be more willing to cooperate; when they believe that their interests and goals are discordant (negative outcome interdependence), they may have more malicious expectations about each other and be more inclined to compete. Importantly, great power relations often involve “mixed-motive” situations (Komorita and Parks, 1995) where both parties have motivations to both cooperate *and* compete (see also Halevy et al., 2006). War or peace thus critically depends upon how the citizens and leaders of great powers perceive their interdependence.

Extant behavioral and psychological research on preemptive strikes at the interpersonal level has revealed that greater perceived risk of being attacked *increases* the frequency of preemptive attacks, while the feeling of hope *decreases* strike rates (Halevy, 2017). At the intergroup level, preemptive strikes even occur between artificial groups (Böhm et al., 2016; cf. Mifune et al., 2017), consistent with the tendency for people to maintain schema-based outgroup mistrust, expecting them to be more competitive, dishonest, and even hostile than individuals (Insko and Schopler, 1998). We suspect that the subjective perception of intergroup relations plays a critical role in evaluating the likelihood and severity of out-group threat, and subsequent decisions to engage in preemptive defensive aggression. In two studies based on a two-person, incentivized decision task, the Preemptive Strike Game (PSG; Simunovic et al., 2013), we explore how perceived great power relations shape defensive preemptive aggression between citizens of those great powers.

## Study 1

We designed a three-country natural experiment to examine preemptive strikes between American (*N* = 115), Chinese (*N* = 106), and Japanese college students (*N* = 99). We predicted that preemptive strikes would be more pronounced in the intergroup than intragroup context (*hypothesis 1*), and that preexisting subjective perceptions of the nature of bilateral great power interdependence (positive/cooperative vs. negative/competitive) would shape the frequency of preemptive strikes against participants from the other nations (*hypothesis 2*). We also hypothesized that preexisting national stereotypes would drive aggression (*hypothesis 3*). According to the stereotype content model (SCM; Fiske et al., 2007), the degree of threat posed by another social group is assessed based upon its perceived intentions (warmth) and strength (competence). These socially conditioned stereotypes, therefore, should also shape the frequency of preemptive strikes. Lastly, we implemented further measures to explore whether any preemptive strikes taken were more defensively or offensively driven.

## Materials and Methods

### Participants

Participants were recruited from an American (Mid-Atlantic region), a Chinese (Southeastern region), and two Japanese (Tokyo area) universities. They were undergraduates enrolled in psychology courses, participated voluntarily (with written consent), and received monetary compensation. Sample sizes were decided prior to data collection, based on prior power analyses (small to medium effect sizes expected; power ≥ 0.80) and funding.

One hundred and twenty-one American participants completed our experiment. Six were excluded for failing to understand the PSG rules (they failed to recall the correct pay-off rules in the post-PSG questionnaire). One hundred and fifteen American participants (52% male) were therefore included in the final analyses. Their ages ranged from 18 to 29 (*M* = 18.99 years, *SD* = 1.22), and they came from 13 U.S. States.

One hundred and twenty-one Chinese participants completed our experiment. Fifteen were excluded for failing to understand the PSG rules. As a result, 106 Chinese participants (34% male) were included in the final analyses. Their ages ranged from 18 to 25 (*M* = 19.52 years, *SD* = 1.04), and they came from 17 Chinese provinces.

One hundred and two Japanese participants completed our experiment. Three were excluded for failing to understand the PSG rules. Ninety-nine Japanese participants (50% male) were therefore included in the final analyses. Their ages ranged from 18 to 23 (*M* = 19.19 years, *SD* = 1.31), and they came from 27 Japanese counties.

Importantly, gender did not shape preemptive strike rates (*ps* > 0.250) in any country; the disparity of gender composition in the three national samples should not, therefore, affect our key findings. We also compared results with or without excluding participants who had not understood the task; the major findings remained robust.

### Materials and Procedures

Participants completed a survey online 1 week prior to the PSG experiment. It assessed their preexisting perceptions about the two bilateral relationships that included their own country, their preexisting national stereotypes of the American, Chinese, and Japanese people, as well as other personality and demographic variables of interest. One week later, participants came to the laboratory and completed the PSG experiment. They also filled out a post-experiment survey tapping into the drivers of their PSG decisions. The original survey and experimental materials were written in English and translated into Chinese and Japanese by bilingual researchers.

#### Pre-PSG Survey

##### Preexisting perceptions of bilateral relations

Participants were asked to report how they viewed bilateral relations between their own country and the other two countries. Items included perceived competition (“*Do you see the following bilateral relations as more competitive or cooperative?*” 1 = *Very competitive*, 7 = *Very cooperative*), likelihood of future military conflict (“*How likely is a military conflict between [your country] and the following countries in the next 10 years?*” 1 = *Very unlikely*, 7 = *Very likely*), and optimism about future bilateral relations (“*Do you feel more pessimistic or optimistic about the future of the following bilateral relations?*” 1 = *Very pessimistic*, 7 = *Very optimistic*). Perceived competition was reverse coded such that higher scores indicate greater competition between the two countries.

##### National stereotypes

Participants reported their general impressions of people from the United States, China, and Japan, including perceived warmth and competence. Specifically, participants rated to what extent Americans, Chinese, and Japanese possess 6 traits on a 7-point Likert scale (1 = *Not at all*, 7 = *Very much*). Trait adjectives, validated in previous research (Chen et al., 2016), assessing warmth included *likeable*, *friendly*, and *nice*; adjectives assessing competence included *competent*, *intelligent*, and *capable*. Adjective order was randomized. Cronbach’s alphas for each measure ranged from 0.84 to 0.90 in the American sample, from 0.81 to 0.89 in the Chinese sample, and from 0.66 to 0.88 in the Japanese sample (only the alpha for the perceived warmth of Japanese people themselves was below 0.70). Average scores across the adjectives for warmth and competence were therefore calculated.

Bivariate correlations between warmth and competence for each national target ranged from 0.24 to 0.53 in the American sample, from 0.51 to 0.58 in the Chinese sample, and from 0.24 to 0.33 in the Japanese sample. These correlations were small to moderate, suggesting that warmth and competence were related but distinct.

#### PSG Lab Experiment

We used Qualtrics survey software to program and administer the experiment in all three languages and countries. Participants completed PSG tasks privately in cubicles. Upon arrival, they received cash for participation (United States: 4 dollars; China: 14 yuan; Japan: 300 yen).^1^ Participants were also informed that they might receive additional cash rewards depending upon their PSG decisions.

##### Red-button PSG

In this key decision task, two paired participants make decisions on separate computers. Within 30-s, each decides whether to click a red button in the center of their computer screen or not. Their decisions have monetary consequences: if neither participant clicks the button, each receives the maximum cash reward (United States: 14 dollars; China: 46 yuan; Japan: 1,000 yen). Otherwise, the participant who clicks first pays a small cost (United States: receive 12 of 14 dollars; China: receive 41 of 46 yuan; Japan: receive 900 of 1,000 yen), while the other receives nothing, suffering a great monetary loss. Importantly, once the red button is clicked, any later clicks lose their effect; so retaliation is not possible. As a result, clicking the red button is an act of preemptive aggression, which may reflect either a defensive desire to eliminate a perceived threat to one’s material well-being, or an offensive desire to harm the other participant.

**FIGURE 1:**
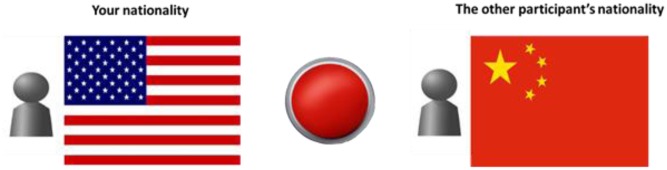
A stimulus for a PSG decision: the United States–China PSG. American participants decided whether to click the red button within 30-s, facing an anonymous Chinese participant.

Following a practice session, each participant made red-button decisions three times, facing a different participant from the United States, China, and Japan. The sequence was counterbalanced. In each round, a 5-s countdown (preparation time) was given before the 30-s countdown to make the red-button decision. No personal information was disclosed about either participant, except for their nationalities, indicated with a national flag (**Figure 1**). Due to their different locations and time zones, decisions were not made in real-time, and no immediate outcome feedback was provided after each round. After all data were collected, we randomly paired participants’ decisions in a random round to calculate their cash rewards. Our procedure ensured the anonymity of participants’ decisions.

##### Red-blue-button PSG

After the red-button PSG, participants who clicked the red button were provided with an additional option to switch their red-button decisions to clicking a blue button instead. Unlike the red button, clicking the blue button incurs a small and identical cost to *both* participants (United States: lose 2 dollars, receive 12 dollars; China: lose 5 yuan, receive 41 yuan; Japan: lose 100 yen, receive 900 yen), rather than primarily harming the other participant. Switching to the blue button therefore indicates defensive aggression, whereas sticking to the red button indicates offensive aggression (Simunovic et al., 2013).

Decisions in this red-blue-button PSG also had monetary consequences; switching to clicking the blue button could change the final payoffs for both participants. For instance, if a participant who clicked the red button first in the red-button PSG later decides to click the blue button instead, both participants in this round would receive identical payoffs, with no one losing everything. In short, we paid participants cash rewards considering the results of this follow-up red-blue-button PSG. Participants made the red-blue-button decisions for each of the three rounds in which they previously clicked the red button.

For more details about the two PSG tasks, including the original instructions in each language, please see the Supplemental Materials.

#### Post-PSG Survey

After making all PSG decisions, participants answered additional survey questions and completed extra decision tasks.

##### Expectations about the other participant’s red-button decision

In strategic interaction, the decision to cooperate or compete in situations involving a possible conflict of interest is strongly influenced by expectations about what the other player will do (for a review, see Balliet and Van Lange, 2013). To better understand the proximal drivers of preemptive strikes, we also asked participants to indicate what percentage of the American, Chinese, and Japanese participants they expected to click the red button when playing the red-button PSG against them.

##### Hypothetical unilateral PSG

In addition to the incentivized, red-blue-button PSG, we also implemented a hypothetical PSG to assess each participant’s inclinations to engage in offensive aggression against specific others. Specifically, each participant imagined a red-button PSG in which *only* he or she has the option to click the red button; the other player cannot do anything so there is no threat of material harm. The payoff rules were same as the red-button PSG: clicking the red button incurs a small loss to oneself but causes the other participant to lose everything. Each participant indicated how likely he or she would be to click the button facing an American, a Chinese person, and a Japanese person. The responding scale was a 7-point Likert scale (1 = *Very unlikely*, 7 = *Very likely*). Given that the other participant has no capability to attack, the decision to click the button indicates purely offensive or non-provoked aggression.

For other measures in the Pre-PSG and Post-PSG surveys, please see the Supplemental Materials.

## Results

### Comparisons of Preexisting Perceptions of Bilateral Relations

**Table 1** displays descriptive statistics of preexisting perceptions of the nature of each pair of bilateral relations for participants from each country. We performed repeated measures ANOVAs^2^ to compare preexisting perceptions of various bilateral relations in each of the three samples.

**Table 1:** Preexisting perceptions of bilateral relations reported by American, Chinese, and Japanese participants.

	United States–China			United States–Japan			China-Japan		
			
	Compe.	Conf.	Optim.	Compe.	Conf.	Optim.	Compe.	Conf.	Optim.
American	3.59 (1.74)	3.66 (1.36)	4.35 (1.28)	3.01 (1.50)	3.11 (1.37)	4.67 (1.15)			
Chinese	3.71 (1.79)	3.75 (1.55)	4.84 (1.06)				4.07 (1.58)	4.45 (1.54)	4.00 (1.22)
Japanese				2.29 (1.25)	3.10 (1.42)	4.24 (1.29)	4.34 (1.29)	4.73 (1.48)	2.90 (1.25)


*Ratings in each row are from American, Chinese and Japanese participants, respectively. Compe. = Perceived competition; Conf. = Projected likelihood of military conflict; Optim. = Optimism about future bilateral relations. Mean scores are reported outside parentheses; standard deviations are reported in parentheses.*

For American participants, perceptions of United States–China and United States–Japan relations differed significantly with respect to perceived competition (*F*[1,114] = 11.41, *p* = 0.001, partial η^2^ = 0.091), likelihood of future military conflict (*F*[1,114] = 17.84, *p* < 0.001, partial η^2^ = 0.135), and optimism about future bilateral relations (*F*[1,114] = 7.75, *p* = 0.006, partial η^2^ = 0.064). Pairwise comparisons indicated that United States–China relations were perceived as more competitive (*M*_difference_ = 0.58, *p* = 0.001, 95% CI = [0.24, 0.92]) and likely to be conflictual (*M*_difference_ = 0.55, *p* < 0.001, 95% CI = [0.29, 0.80]) than United States–Japan relations. Additionally, American participants felt less optimistic about United States–China relations than United States–Japan relations (*M*_difference_ = -0.32, *p* = 0.006, 95% CI = [-0.55, -0.09]).

For Chinese participants, perceptions of China–United States and China-Japan relations also differed significantly with respect to perceived competition (*F*[1,105] = 4.28, *p* = 0.041, partial η^2^ = 0.039), likelihood of future military conflict (*F*[1,105] = 29.65, *p* < 0.001, partial η^2^ = 0.220), and optimism about future bilateral relations (*F*[1,105] = 57.58, *p* < 0.001, partial η^2^ = 0.354). Pairwise comparisons indicated that China-Japan relations were perceived as more competitive (*M*_difference_ = 0.36, *p* = 0.041, 95% CI = [0.01, 0.70]) and likely to be conflictual (*M*_difference_ = 0.71, *p* < 0.001, 95% CI = [0.45, 0.96]) than China–United States relations. Additionally, Chinese participants felt less optimistic about China-Japan relations than China–United States relations (*M*_difference_ = -0.84, *p* < 0.001, 95% CI = [-1.06, -0.62]).

For Japanese participants, perceptions of Japan-China and Japan–United States relations also differed significantly with respect to perceived competition (*F*[1,98] = 161.40, *p* < 0.001, partial η^2^ = 0.622), likelihood of future military conflict (*F*[1,98] = 113.95, *p* < 0.001, partial η^2^ = 0.538), and optimism about future bilateral relations (*F*[1,98] = 70.51, *p* < 0.001, partial η^2^ = 0.418). Pairwise comparisons indicated that Japan-China relations were perceived as more competitive (*M*_difference_ = 2.05, *p* < 0.001, 95% CI = [1.73, 2.37]) and likely to be conflictual (*M*_difference_ = 1.63, *p* < 0.001, 95% CI = [1.32, 1.93]) than Japan–United States relations. Additionally, Japanese participants felt less optimistic about Japan-China relations than Japan–United States relations (*M*_difference_ = -1.34, *p* < 0.001, 95% CI = [-1.66, -1.03]).

### Comparisons of Preexisting National Stereotypes

**Table 2** displays descriptive statistics for reported national stereotypes. We also performed repeated measures ANOVAs to compare preexisting national stereotypes in each of the three samples.

**Table 2:** National stereotypes reported by American, Chinese, and Japanese participants.

	Warmth			Competence		
		
	American	Chinese	Japanese	American	Chinese	Japanese
American	4.46 (0.92)	4.23 (1.00)	4.48 (1.07)	4.99 (0.99)	5.35 (0.94)	5.20 (1.00)
Chinese	4.60 (0.99)	5.00 (1.11)	4.04 (1.22)	5.57 (1.02)	5.58 (0.98)	5.26 (1.03)
Japanese	5.15 (1.03)	2.93 (0.98)	4.77 (0.99)	5.14 (1.02)	4.40 (1.27)	4.50 (1.05)


*Ratings in each row are from American, Chinese, and Japanese participants, respectively. Ratings in each column are national stereotypes about Americans, Chinese, and Japanese people. Mean scores are reported outside parentheses; standard deviations were reported in parentheses.*

For American participants, stereotypes about Americans, Chinese, and Japanese differed significantly with respect to perceived warmth (*F*[1.82,207.91] = 4.72, *p* = 0.011, partial η^2^ = 0.040) and competence (*F*[1.88,214.56] = 8.39, *p* < 0.001, partial η^2^ = 0.069). Pairwise comparisons indicated that Chinese were perceived as colder than both Americans (*M*_difference_ = -0.23, *p* = 0.044, 95% CI = [-0.46, 0.00]) and Japanese (*M*_difference_ = -0.26, *p* = 0.004, 95% CI = [-0.44, -0.07]). On the other hand, Chinese were perceived as more competent than Americans (*M*_difference_ = 0.36, *p* < 0.001, 95% CI = [0.14, 0.59]) but not more than Japanese (*M*_difference_ = 0.15, *p* = 0.172, 95% CI = [-0.04, 0.34]).

For Chinese participants, stereotypes about Americans, Chinese, and Japanese differed significantly with respect to perceived warmth (*F*[1.78,187.61] = 38.65, *p* < 0.001, partial η^2^ = 0.269) and competence (*F*[2,210] = 10.04, *p* < 0.001, partial η^2^ = 0.087). Pairwise comparisons indicated that Japanese were perceived as colder than both Chinese (*M*_difference_ = -0.96, *p* < 0.001, 95% CI = [-1.27, -0.65]) and Americans (*M*_difference_ = -0.56, *p* < 0.001, 95% CI = [-0.82, -0.31]). Japanese also were perceived as less competent than Chinese (*M*_difference_ = -0.32, *p* = 0.001, 95% CI = [-0.53, -0.11]) and Americans (*M*_difference_ = -0.31, *p* < 0.001, 95% CI = [-0.49, -0.14]).

For Japanese participants, stereotypes about Americans, Chinese, and Japanese also differed significantly with respect to perceived warmth (*F*[1.88,184.58] = 152.23, *p* < 0.001, partial η^2^ = 0.608) and competence (*F*[2,196] = 18.60, *p* < 0.001, partial η^2^ = 0.160). Pairwise comparisons indicated that Chinese were perceived as colder than both Americans (*M*_difference_ = -2.23, *p* < 0.001, 95% CI = [-2.56, -1.90]) and Japanese (*M*_difference_ = -1.84, *p* < 0.001, 95% CI = [-2.14, -1.55]). On the other hand, Americans were perceived as more competent than both Chinese (*M*_difference_ = 0.75, *p* < 0.001, 95% CI = [0.44, 1.06]) and Japanese (*M*_difference_ = 0.64, *p* < 0.001, 95% CI = [0.33, 0.95]).

In all three countries, negative perceptions of bilateral relations (i.e., competition and conflict, but not optimism) were negatively correlated with warmth toward people of that country, except that Chinese and Japanese warmth toward Americans was not significantly correlated with any perceived qualities of United States–China or United States–Japan relations. On the other hand, perceptions of bilateral relations did not consistently correlate with the perceived competence of foreigners. See the Supplemental Materials for these correlations.

### Preemptive Strikes under Each Condition and in Each Country

**FIGURE 2:**
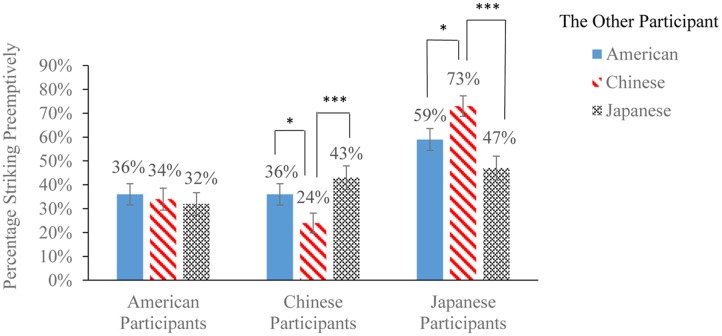
Percentages of American, Chinese, and Japanese participants striking preemptively (±1 SE) in the red-button PSG, facing a different participant from each of the three countries. All decisions were incentivized. ^∗^*p* < 0.05; ^∗∗∗^*p* < 0.001.

**Figure 2** displays the frequencies of red-button decisions (i.e., preemptive strikes) under each PSG condition. To test the main effect of the other participant’s nationality, we performed a repeated measures logistic regression (Generalized Estimating Equations; GEE), thus controlling for individual attacking proclivities across all three PSG decisions. The regression revealed, unexpectedly, that the other participant’s nationality did not influence the rates of American participants’ PSG attacks, χ^2^ = 0.69, *df* = 2, *p* > 0.250. As illustrated in **Figure 2**, the proportions of American participants making preemptive strikes was almost identical across all three conditions. Consistent with this finding, a repeated measures ANOVA also found that American participants expected a similar percentage of other participants in each of the three countries (United States: *M* = 51%, 95% CI = [46%, 56%]; China: *M* = 52%, 95% CI = [47%, 57%]; Japan: *M* = 51%, 95% CI = [46%, 55%]) to make preemptive strikes against them, *F*(1.38,157.29) = 0.28, *p* > 0.250, partial η^2^ = 0.00.

As expected, however, the other participant’s nationality significantly affected Chinese participants’ rates of PSG attacks, χ^2^ = 18.28, *df* = 2, *p* < 0.001. Planned comparisons^3^ indicated that, consistent with hypothesis 1, preemptive strikes were more frequent in the China-Japan PSG (*M*_difference_ = 20%, *p* < 0.001, 95% CI = [11%, 29%]) and the China–United States PSG (*M*_difference_ = 12%, *p* = 0.026, 95% CI = [2%, 22%]) than the within-country (China-China) PSG (**Figure 2**). A repeated measures ANOVA found that Chinese participants expected more Japanese (*M* = 45%, 95% CI = [39%, 50%]) and American (*M* = 46%, 95% CI = [40%, 52%]) participants to preemptively strike them than other Chinese participants would (*M* = 34%, 95% CI = [28%, 39%]), *F*(2,210) = 14.20, *p* < 0.001, partial η^2^ = 0.12.

Likewise, the other participant’s nationality significantly shaped Japanese participants’ PSG rates of attack, χ^2^ = 23.23, *df* = 2, *p* < 0.001. Consistent with hypothesis 1, preemptive strikes were more frequent in the Japan-China PSG (*M*_difference_ = 25%, *p* < 0.001, 95% CI = [15%, 40%]) and the Japan–United States PSG (*M*_difference_ = 11%, *p* = 0.037, 95% CI = [2%, 20%]) than the within-country (Japan-Japan) PSG (**Figure 2**). A repeated measures ANOVA revealed that Japanese participants expected more Chinese participants (*M* = 62%, 95% CI = [57%, 68%]) to preemptively strike them than American participants (*M* = 57%, 95% CI = [52%, 63%]) and other Japanese participants (*M* = 56%, 95% CI = [50%, 62%]), *F*(1,98) = 4.36^4^, *p* = 0.039, partial η^2^ = 0.04.

### The Impact of Bilateral Relations and National Stereotypes on Preemptive Strikes

A GEE logistic regression was conducted to examine how preexisting perceptions of bilateral relations and national stereotypes shaped PSG aggression. First, the decision to click the red button in the two intergroup PSGs (e.g., United States–China and United States–Japan PSGs for American participants) was regressed onto perceived competition, the likelihood of military conflict, and optimism about future bilateral relations, for each of the three national samples separately. As illustrated in **Table 3**, for American participants, optimism about future bilateral relations significantly *reduced* the likelihood of making preemptive strikes against foreigners, *B* = -0.41, odds ratio Exp (*B*) = 0.66, *p* = 0.015, 95% CI = [0.47, 0.92]. For Chinese participants, greater projected likelihood of military conflict significantly *increased* the odds of making preemptive strikes against foreigners, *B* = 0.20, Exp (*B*) = 1.23, *p* = 0.038, 95% CI = [1.01, 1.49]. Similarly, for Japanese participants perceived competitiveness of bilateral relations significantly *increased* the odds of preemptive strikes, *B* = 0.34, Exp (*B*) = 1.40, *p* = 0.006, 95% CI = [1.10, 1.79]. Taken together, these results support hypothesis 2 that greater perceived great power competition promotes preemptive strikes against citizens of those great powers.

**Table 3:** Regressing preemptive strikes onto preexisting perceptions about bilateral relations.

	The decision to click the red button in the inter-group PSGs		
	
	American participants	Chinese participants	Japanese participants
Perceived competition	1.04 [0.83, 1.31]	1.07 [0.89, 1.28]	1.40^∗∗^ [1.10, 1.79]
Likelihood of conflict	0.78 [0.60, 1.01]	1.23^∗^ [1.01, 1.49]	0.82 [0.64, 1.06]
Optimism	0.66^∗^ [0.47, 0.92]	1.04 [0.79, 1.37]	0.89 [0.66, 1.20]


*The red-button decisions in the two inter-group PSGs were regressed (GEE logistic) onto three perceptions of bilateral relations simultaneously. Each column reports regression results for American, Chinese, and Japanese participants, respectively. Likelihood of conflict = Projected likelihood of military conflict; Optimism = Optimism about future bilateral relations. Odds ratios Exp (B) are reported in the table. 95% CIs are reported in [square brackets]. ^∗^*p* < 0.05; ^∗∗^*p* < 0.01.*

Second, to test the role of preexisting national stereotypes in shaping aggression, the intergroup PSG decisions were regressed onto the perceived warmth and competence of the two foreign peoples. For American participants, neither warmth (*B* = -0.16, Exp [*B*] = 0.85, *p* > 0.250, 95% CI = [0.63, 1.15]) nor competence (*B* = -0.25, Exp [*B*] = 0.78, *p* = 0.123, 95% CI = [0.57, 1.07]) was a significant predictor of preemptive strikes against Chinese and Japanese. By contrast, for both the Chinese and Japanese participants, greater warmth toward the two groups of foreigners significantly *reduced* preemptive strikes in the intergroup PSG (China: *B* = -0.27, Exp [*B*] = 0.76, *p* = 0.028, 95% CI = [0.60, 0.97]; Japan: *B* = -0.29, Exp [*B*] = 0.75, *p* = 0.002, 95% CI = [0.62, 0.90]). Perceived competence, on the other hand, did not reliably predict intergroup PSG aggression (China: *B* = -0.20, Exp [*B*] = 0.82, *p* = 0.135, 95% CI = [0.63, 1.07]; Japan: *B* = -0.16, Exp [*B*] = 0.85, *p* = 0.223, 95% CI = [0.65, 1.10]). Hypothesis 3 is thus partly supported.^5^

Last, we also regressed the expectation of the foreign participant’s red-button decision onto perceived bilateral relations as well as national stereotypes. For American participants, neither perceived bilateral relations nor national stereotypes reliably predicted the expected rate of outgroup preemptive strikes (*ps* ≥ 0.10). For Chinese participants, greater projected likelihood of military conflict increased the expected rate of outgroup attacks, *B* = 3.40, *p* = 0.037, 95% CI = [0.21, 6.58], whereas greater warmth toward Japanese and Americans reduced the expectation of outgroup attacks, *B* = -4.39, *p* = 0.047, 95% CI = [-8.72, -0.06]. For Japanese participants, greater perceived bilateral competition increased the expected rate of outgroup attacks, *B* = 3.46, *p* = 0.021, 95% CI = [0.53, 6.40], whereas greater warmth toward Chinese and Americans reduced the expectation of outgroup attacks, *B* = -3.45, *p* = 0.017, 95% CI = [-6.28, -0.62]. In all three countries, expected rates of outgroup preemptive strikes were positively associated with the odds that a participant would click the red button in the intergroup PSGs (United States: *B* = 0.05, Exp [*B*] = 1.05, *p* < 0.001, 95% CI = [1.03, 1.07]; China: *B* = 0.06, Exp [*B*] = 1.06, *p* < 0.001, 95% CI = [1.05, 1.08]; Japan: *B* = 0.04, Exp [*B*] = 1.04, *p* < 0.001, 95% CI = [1.03, 1.06]).

### Preemptive Strikes Are Primarily Defensive

**FIGURE 3:**
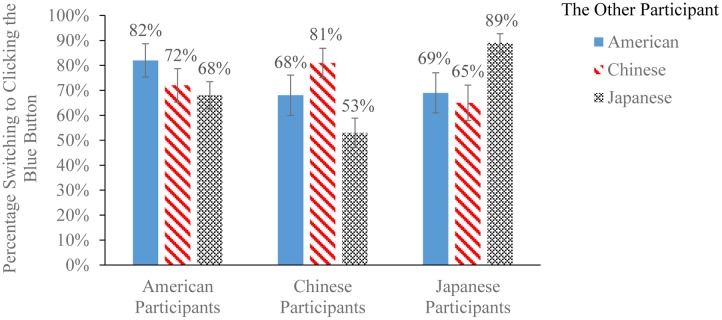
Percentages switching from offensive to defensive preemptive strikes among American, Chinese, and Japanese participants (±1 SE) in the red-blue-button PSG, facing a different participant from each of the three countries. Participants decided to stick to their previous red-button decisions (offensive preemptive strikes) or switch to clicking a blue button instead (defensive preemptive strikes). Decisions were incentivized. Results were based on participants who correctly identified the pay-off rules for this task (United States: *N* = 33; China: *N* = 45; Japan: *N* = 72).

As illustrated in **Figure 3**, in the red-blue-button PSG, the majority of participants ( > 50%) in each country switched from their previous red-button decisions to clicking the blue button, regardless of the other participant’s nationality. This provides initial evidence that preemptive strikes in our experiment were primarily defensive. However, we also noticed that the percentage of participants switching their decision was greater in the intragroup context than in the intergroup context, suggesting some degree of intergroup bias. Additionally, both Chinese and Japanese participants were more inclined to stick to their red-button decisions when facing each other than when facing Americans (**Figure 3**), consistent with the fact that China-Japan relations were perceived by both the Chinese and Japanese as more conflictual than United States–China or United States–Japan relations. But overall, the frequency of offensive aggression was quite low in all three countries. For instance, in China, only 15% of all 106 participants chose to stick to the red button when facing Japanese; in Japan, only 24% of all 99 participants chose to stick to the red button when facing Chinese.

Likewise, as illustrated in **Table 4**, participants in all three countries reported low likelihoods of initiating offensive preemptive strikes in the hypothetic unilateral PSG, regardless of the other participant’s nationality. We further performed one-sample *t*-tests to examine whether participants’ reported likelihoods of offensive aggression against each national group were significantly different from the neutral point (*4* = *Undecided)*; the results indicated that the reported likelihood of offensive aggression was significantly lower than the neutral point under each PSG condition and in each of the three samples (*ps* < 0.001; all CIs of mean differences excluded the point of zero).

**Table 4:** Reported likelihood of offensive aggression in a hypothetical unilateral PSG.

	Likelihood of clicking the red button when the other participant cannot attack		
	
	The other: American	The other: Chinese	The other: Japanese
American	2.50 (1.93)	2.50 (1.92)	2.47 (1.90)
Chinese	2.43 (1.60)	1.95 (1.38)	2.80 (1.92)
Japanese	2.11 (1.82)	2.25 (1.91)	2.21 (1.95)


*Reported likelihoods in each row were from American, Chinese and Japanese participants, respectively. Answers were on a 7-point scale (*1* = *Very unlikely*, *7* = *Very likely*). Mean scores are reported outside parentheses; standard deviations were reported in parentheses. This question was hypothetical and not incentivized.*

## Discussion

Consistent with our hypotheses, preexisting perceptions of the nature of each bilateral relationship and national stereotypes both shaped intergroup preemptive strike rates among Chinese and Japanese college students. Importantly, we also found that preemptive strikes were driven more by defensive than offensive aggression. A proximal and defensive driver of intergroup preemptive strikes appears to be the expectation of the outgroup member’s imminent aggression. As “security dilemma” theorists suggest, fear can lead to war (see also Böhm et al., 2016).

On the other hand, the American college students in our sample did not exhibit the intergroup bias that we predicted. It could be that our sample of East Coast American students were more individualistic, liberal, and motivated not to appear racist than our Chinese and Japanese students were (Henrich et al., 2010). Additionally, our American students may view great power relations as more indeterminate than their Chinese and Japanese counterparts. In Study 1, only American students’ optimism about the two bilateral relationships reduced intergroup preemptive strike rates, consistent with the role of hope in mitigating preemptive aggression between individuals (Halevy, 2017), as well as promoting conflict resolution between rival countries (Cohen-Chen et al., 2014). However, such rosy findings may not apply to older and more conservative Americans who may perceive great power relations to be more contentious.

In the next study, we address the issue of range restriction of age and ideologies in our American student sample. Additionally, Study 1’s natural experiment design prevents a deeper exploration of causal mechanisms. The next study further explores the precise causal mechanisms linking perceived great power interdependence to intergroup preemptive aggression.

## Study 2

We designed a randomized experiment to better understand causal mechanisms and manipulate a more diverse sample of adult Americans’ perceptions of United States–China relations, using real CNN expert interviews that framed United States–China economic interdependence as either more positive or more negative. We chose to manipulate specifically *economic* interdependence for two reasons. First, it is highly *salient*, as the United States media frequently covers United States–China trade, investment, currency, and jobs, increasing the mundane realism of our experiment. United States–China economic relations were also a central theme of the Trump presidential campaign, when study 2 was run. Second, manipulating *economic* interdependence is *plausible*, as strong cases can be made either that it is positive (e.g., vast mutual absolute gains from trade and investment) or negative (e.g., currency manipulation, unfair trade practices, job losses).

We predicted both that more positive-sum news reports would *decrease* preemptive American strikes against Chinese participants (*hypothesis 4*), and that more zero/negative-sum news reports would amplify mistrust and anger toward China, *increasing* preemptive American strikes against Chinese (*hypothesis 5*). Mistrust and anger are psychological consequences of intergroup threat (Williams, 2001; Cottrell and Neuberg, 2005). These negative feelings are therefore likely to promote preemptive strikes (Halevy, 2017).

## Materials and Methods

### Participants

Participants were recruited from Amazon MTurk’s online marketplace for employers and workers. Sample size was determined based on prior power analyses (small to medium effect sizes expected; power ≥ 0.80). One hundred and forty American citizens completed our online experiment (with written consent). Eleven failed to pass a memory test about the news report’s content; four failed a test about the PSG rules. These 13 participants were excluded from the final analyses presented here. However, when we compared results with or without excluding these 13 participants; our key findings remained robust.

The final sample included 127 American citizens (51% male). Their ages ranged from 23 to 70 (*M* = 40.24, *SD* = 11.83). The racial distribution of the sample was 84.3% Caucasian American, 5.5% African American, 4.7% Hispanic American, 3.9% Asian American, and 0.8% Native American. The sample was well-educated, with high school (20.5%), college (66.9%), and post-graduate degrees (11.8%). Participants came from 31 states. The sample included 45.7% Democrats, 18.1% Republicans, 33.9% Independents, and 2.4% Libertarians. Ideologically, the sample was slightly liberal (*M* = 3.24, *SD* = 1.58; scale midpoint = 4).

### Materials and Procedures

Participants completed a 20-min online survey. The randomized experiment began with a practice session where each participant made hypothetical red-button PSG decisions facing another American. Following the PSG practice, participants were asked to watch a 2–3 min CNN expert interview about IR. They then answered a number of survey questions and completed two incentivized intergroup PSG decisions.

#### Video Stimuli

Participants were randomly assigned to watch one of two CNN expert interviews: one (with former Treasury Secretary Hank Paulson) portrayed United States–China economic interdependence as more positive, while the other (with former Goldman Sachs partner Peter Kiernan) portrayed it as more negative. Both video clips were edited from real CNN reports, and were developed and validated in a prior study (see Supplemental Materials). “Kiernan” interview clip link: https://www.youtube.com/v/vRmXNOIEbP0. “Paulson” interview clip link: https://www.youtube.com/v/rLKDpB7ORyk.

#### Perceptions of the News Report

Immediately after watching one of the two randomly assigned news clips, participants were asked, “*To what extent does this video clip describe United States–China relations as competitive or as cooperative?*” Responses were reverse coded as 1 = *Extremely cooperative*, 4 = *Neutral*, 7 = *Extremely competitive*. To control for noise in the real world video clips, participants also rated to what extent they viewed the “person interviewed” as (1) *likeable*, (2) *credible*, and (3) *attractive*, and the “news report itself” as (1) *professional*, (2) *engaging*, (3) *balanced*, and (4) *stimulating*. The responses were on a 7-point Likert scale (1 = *Not at all*, 7 = *Completely*). The order of adjectives within each set was randomized.

#### Trust and Anger Toward China

Participants then reported their attitudinal trust in various groups of peoples (“*To what extent do you trust the following groups of people?*”) and governments (“*To what extent do you trust the following governments?*”), including the Chinese people and government. Responses were on 7-point Likert scales (1 = *Strongly distrust*, 7 = *Strongly trust*). Participants were also asked about China’s rise: “*To what extent should Americans feel in the following ways about China’s possible rise?*” *Angry* and *happy* were presented in randomized order, and were rated on 7-point Likert scales (1 = *Not at all*, 7 = *Extremely*). Only angry was analyzed in this study.

#### Intergroup Red-Button PSG

Each American participant then completed two incentivized red-button PSG decisions facing a Chinese and a Japanese person (the order of nationality was counterbalanced). As in Study 1, PSG decisions were not made in real-time. After all data were collected, we randomly paired participants’ decisions with those made by Chinese and Japanese participants in Study 1.^6^ We paid each participant based on the randomly paired decisions from a random round.

We also used Qualtrics survey software to program and implement this online experiment. In each PSG round, both participants’ national flags were displayed. Task procedures and experimental materials were identical to Study 1’s red-button PSG. However, monetary incentives changed: following a recent MTurk PSG study (Halevy, 2017), all MTurkers were paid 150 cents for participating, as well as a variable “bonus”: 300 cents if neither participant attacks, 250 cents if a participant attacks first, which also makes the other participant lose 250 cents (and receive just 50 cents). Final total payments of between 200 and 450 cents were later transferred to each MTurker privately via MTurk’s online platform; anonymity was ensured.

#### Post-PSG Survey

Finally, participants answered questions tapping into their motivations for their PSG decisions and about their demographic backgrounds and personalities. As in study 1, we asked participants to indicate how frequently they expected Chinese and Japanese participants to make preemptive strikes against them; we also assessed their offensive intentions in the hypothetical unilateral PSG facing Chinese and Japanese participants. For other measures in the Post-PSG survey, please see the Supplemental Materials.

## Results

### Manipulation Check

A one-way ANOVA revealed a massive main effect of media framing on perceived United States–China competition, *F*(1,125) = 192.77, *p* < 0.001, partial η^2^ = 0.61. As expected, participants randomly assigned to watch the more zero/negative-sum interview (“Kiernan”: *M* = 6.31, 95% CI = [6.06, 6.55]) viewed United States–China relations to have been depicted as much more competitive than those who watched the more positive-sum interview (“Paulson”: *M* = 3.39, 95% CI = [3.05, 3.78]). A one-way MANOVA also revealed that participants viewed each interviewee’s personality and each video clip’s production value differently, *F*(7,119) = 15.08, *p* < 0.001, partial η^2^ = 0.47. Paulson and his clip (more positive-sum) were generally viewed more favorably than Kiernan and his clip (the Supplemental Materials). We therefore compared our results with or without controlling for these perceptions of the interviewee’s personality and the clip’s production values. It had limited influence on our key analyses.

In addition, a one-way ANOVA found that, as expected, media portrayal of more negative United States–China economic interdependence significantly and substantially *increased* anger toward China’s possible rise (“Kiernan”: *M* = 3.44, *SD* = 1.69; “Paulson”: *M* = 2.32, *SD* = 1.32), *F*(1,125) = 16.96, *p* < 0.001, partial η^2^ = 0.12. However, unexpectedly, a one-way MANOVA found no significant main effect of media framing on trust in the Chinese people (“Kiernan”: *M* = 3.44, *SD* = 1.59; “Paulson”: *M* = 3.76, *SD* = 1.34) and government (“Kiernan”: *M* = 2.35, *SD* = 1.31; “Paulson”: *M* = 2.80, *SD* = 1.32), *F*(2,124) = 1.78, *p* = 0.173, partial η^2^ = 0.03.

### Media Framing of United States–China Economic Interdependence Influences Preemptive Strikes

**FIGURE 4:**
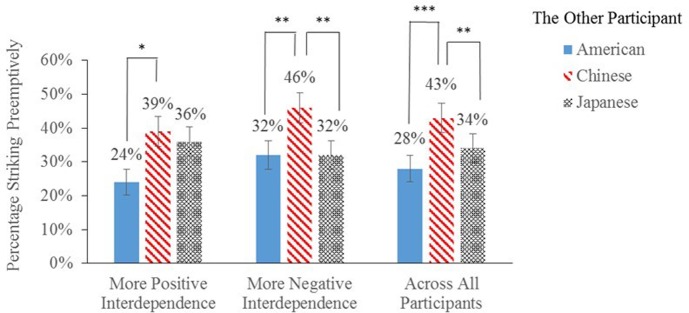
Percentages of American participants striking preemptively (±1 SE) in the red-button PSG, facing a different participant from each of the three countries and under different CNN media manipulations. More positive interdependence = Media portrayal of United States–China economic interdependence as more positive-sum. More negative interdependence = Media portrayal of United States–China economic interdependence as more zero/negative-sum. Participants completed the hypothetical United States–United States PSG before media manipulation, serving as the baseline for the effect of media framing on intergroup preemptive strikes. ^∗^*p* < 0.05; ^∗∗^*p* < 0.01; ^∗∗∗^*p* ≤ 0.001.

We performed a repeated measures GEE regression to examine how the two CNN interviews shaped the rates of preemptive strikes against Chinese persons, controlling for individual attacking proclivities across all three PSG decisions. The regression revealed a significant main effect of the other participant’s nationality, χ^2^ = 15.35, *df* = 2, *p* < 0.001. Planned comparisons indicated that, compared to Study 1’s more range restricted college students, the national Mturk sample of adult Americans made more preemptive strikes against Chinese than against Japanese (*M*_difference_ = 8%, *p* = 0.004, 95% CI = [3%, 13%]) and the other Americans in the practice session (*M*_difference_ = 14%, *p* = 0.001, 95% CI = [6%, 22%]). This effect of the other participant’s nationality also varied across the two experimental conditions (an interaction between media framing and the other participant’s nationality), χ^2^ = 5.40, *df* = 2, *p* = 0.067 (marginal). As illustrated in **Figure 4**, after watching the more zero/negative-sum interview (Kiernan), participants made *more* preemptive strikes against Chinese than against Japanese (*M*_difference_ = 13%, *p* = 0.003, 95% CI = [5%, 21%]). By contrast, after watching the more positive-sum interview (Paulson), preemptive strike rates against Chinese and Japanese participants were the same (*M*_difference_ = 3%, *p* > 0.250, 95% CI = [-4%, 10%]). And consistent with hypothesis 4, portraying United States–China economic interdependence as more positive *reduced* preemptive American strikes against Chinese participants (relative to Japanese participants).^7^

Similarly, a repeated measures ANOVA revealed a marginally significant interaction between media framing and the other participant’s nationality on the expected rate of outgroup preemptive strikes, *F*(1,125) = 3.69, *p* = 0.057, partial η^2^ = 0.03; pairwise comparisons indicated that under both experimental conditions the expected rates of Chinese preemptive strikes (more positive portrayal: *M* = 51%; more negative portrayal: *M* = 59%) were greater than the expected rates of Japanese preemptive strikes (more positive portrayal: *M* = 42%; more negative portrayal: *M* = 44%), but this difference was larger after watching the more negative interview about United States–China interdependence (more positive portrayal: *M*_difference_ = 9%, *p* = 0.001, 95% CI = [4%, 14%]; more negative portrayal: *M*_difference_ = 16%, *p* < 0.001, 95% CI = [11%, 21%]). Across all participants, a GEE regression also found that the expected rate of outgroup preemptive strikes was positively associated with the odds of clicking the red button in the two intergroup PSGs, *B* = 0.11, Exp (*B*) = 1.12, *p* < 0.001, 95% CI = [1.08, 1.15].

### Anger Mediates the Effect of Perceived United States–China Competition on Preemptive Strikes

Generalized estimating equations regressions confirmed that the effect of media framing on preemptive strike rates against Chinese participants was mediated by anger about China’s possible rise (the unique effect of anger on intergroup PSGs: χ^2^ = 4.03, *df* = 1, *p* = 0.045). Specifically, watching the more zero/negative-sum CNN interview (Kiernan) increased anger about China’s rise, in turn increasing the frequency of preemptive attacks against Chinese more than against Japanese (for detailed analyses of this mediation, see Supplemental Materials).

**FIGURE 5:**
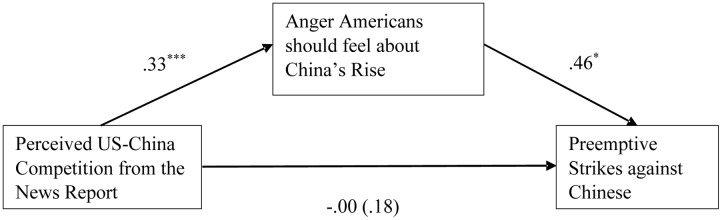
Anger mediated the linkage from perceived United States–China competition to intergroup preemptive strikes against Chinese. Preemptive strikes against Japanese were controlled as a covariate. Preemptive strike was coded as 0 = *No attack* and 1 = *Attack*. The regression involving the binary PSG decision is logistic. For the path from the independent variable (perceived United States–China competition) to the outcome (preemptive strikes against Chinese), the regression coefficient in parentheses did not control for the mediator. All regression coefficients are unstandardized. ^∗^*p* < 0.05; ^∗∗∗^*p* < 0.001.

Across all participants, a mediational analysis using Hayes’s (2013) PROCESS Macro also confirmed that anger mediated the relationship between perceived United States–China competition and the rate of intergroup preemptive strikes (**Figure 5**), indirect effect = 0.15, *p* = 0.041, 95% CI = [0.03, 0.40] (bootstrapping was performed 1000 times). On the other hand, levels of trust in the Chinese people and government did not mediate the main effect of the CNN media manipulation on strike rates; anger appeared to play a more important role in driving intergroup preemptive aggression than did mistrust in the context of media framing and United States–China relations. Hypothesis 5 is thus partly supported.

### Preemptive Strikes Are Primarily Defensive

As in Study 1, participants reported low likelihoods of attacking other participants in the hypothetical unilateral PSG where the other side cannot attack, regardless of the other participant’s nationality (against Chinese: *M* = 2.14, *SD* = 1.94; against Japanese: *M* = 1.86, *SD* = 1.62; both means were significantly below the neutral point of 4, *ps* < 0.001). This also suggests that preemptive strikes were primarily defensive.

## Discussion

Study 2 confirmed both that media portrayals of more positive United States–China economic interdependence *reduced* ordinary Americans’ preemptive strikes against Chinese (but not Japanese), and that more negative media portrayals bolstered American anger toward China’ rise, *increasing* preemptive American strikes against Chinese. The latter is consistent with previous research demonstrating that anger motivates aggressive responses to intergroup threat (Cottrell and Neuberg, 2005). In particular, China’s rise may be considered ethically wrong by some Americans, provoking resentment (Rozin et al., 1999) and aggression.

Importantly, Study 2 results also indicated that American preemptive strikes were motivated more by defensive than offensive aggression. Consistent with Study 1, it appears that fear (the expectation of imminent outgroup aggression) rather than spite was the primary driver of intergroup preemptive aggression.

## General Discussion

“*What made war inevitable was the growth of Athenian power and the fear which this caused in Sparta.*”

(Thucydides [431 B.C.E], 1954).

Nearly 2,500 years ago, Sparta became the dominant power in the Greek world after defeating Athens in the Peloponnesian War. The “Thucydides Trap” refers to the idea that the growth of Athenian power itself made war with Sparta inevitable—and is likely to be repeated by great powers like the United States and China today (Allison, 2015). However, peaceful power transitions such as that from Great Britain to the United States in the 20th century strongly suggest that shifts in the *objective* balance of power itself do not make great power conflict inevitable (Lebow and Valentino, 2009). Instead, this paper argues that the *subjective* perception of negative outcome interdependence is a critical psychological trigger of great power conflict. Across two experiments, we provide the first behavioral evidence that negative perceptions of great power interdependence promote preemptive strikes against citizens from rival countries. Perceived bilateral competition appears to increase the expectation of outgroup aggression, creating the psychological micro foundation for intergroup preemptive aggression.

Importantly, we also found that intergroup preemptive strikes were primarily defensive, even between groups of people who have been socialized to view their bilateral relations negatively (e.g., between Chinese and Japanese) and to hold prejudiced feelings toward one another. This finding is consistent with previous research both at the interpersonal level (Simunovic et al., 2013) and at the intergroup level (Böhm et al., 2016). Humans are motivated more for ingroup defense than for unprovoked outgroup aggression (Böhm et al., 2016; De Dreu et al., 2016; Yamagishi and Mifune, 2016), and tend to avoid zero-sum competition against outgroups (Halevy et al., 2012b; Böhm et al., 2016). Great power conflict is therefore not inevitable, as long as mutual fear and mistrust are restrained.

We did, however, observe remarkably high rates of intergroup preemptive strikes (compared to intragroup preemptive strikes) across both experiments. High intergroup strike rates have not occurred in extant PSG experiments involving artificial groups (cf. Böhm et al., 2016; Mifune et al., 2017), suggesting that the sociopolitical context is a critical driver of intergroup preemptive strike rates. Specifically, compared to when they faced Americans, Chinese and Japanese participants in our PSGs were more likely to attack each other and stick to offensive aggression—even when given the opportunity to act purely defensively. This highlights how socialization in China and Japan today about their past conflicts and current bilateral relations, and mutual prejudices (Brown, 2016) have increased the likelihood of another Sino-Japanese conflict (Cociani, 2016). Geographic proximity and greater contacts between these two East Asian powers make the prospect of preemptive strikes between them even more dangerous.

### Theoretic and Policy Implications

Using the PSG to study the psychological drivers of the security dilemma in great power relations is an example of applying psychological theories (e.g., of conflict, decision making, emotion, and social interdependence) and methods (the PSG, natural and randomized experiments) to the political science subfield of IR. Specifically, while describing the distal structural factors that can lead even benign great powers to defensively back themselves into tragic wars that no one wanted, like World War I, power transition and security dilemma theorists in IR have not explored the proximate psychological triggers (e.g., the perception of social interdependence, the expectation of outgroup offense, and anger) of defensive aggression. This interdisciplinary project begins to fill this gap in our understanding of the micro-level drivers of great power conflict. This project is therefore an example of how social psychology can inform scholarship in other disciplines, and make an applied contribution to the prospects for peace.

Our findings suggest that escaping the “Thucydides Trap” and avoiding preemptive conflict between great powers depends in part upon how Americans, Chinese, and Japanese are socialized (e.g., via education and the media) to feel about each other as people (stereotypes), and to view their bilateral relationships (perceived outcome interdependence). Regrettably, anecdotal evidence strongly suggests that media coverage in the United States, Japan, and China today paints a Hobbesian picture of great power competition—not cooperation. Our results also suggest that the Chinese and Japanese educational systems may do the same, socializing Chinese and Japanese to view each other with suspicion.

Our results thus suggest a critical intervention: combatting stereotypes and promoting more balanced socialization about shared pasts and current bilateral relations. For instance, educational reforms and greater media coverage of positive outcome interdependence between great powers, such as of successful bilateral cooperation and the mutual benefits of trade, would mitigate against preemptive strikes, increasing the probability of great power peace.

China’s rise has led to widespread comparisons to Imperial Germany’s rise a century ago, which prompted a “defensive” arms race and frenzied alliance building—a “security dilemma” contributing to the tragic outbreak of World War I. East Asia today already exhibits strong signs of a similarly “defensive” brew of maritime arms races and alliance politics. The security dilemma addresses distal structural drivers of war, however; it does not examine the more proximal psychological drivers of conflict, or specific forms of aggression like preemptive strikes.

With Donald Trump’s election as President of the United States, in terms of leadership, similarities with the situation prior to World War I have increased. Trump, like Kaiser Wilhelm II of Germany, appears obsessed with respect both for himself personally and for his country. And given his zero-sum view of deal-making and the world more broadly (Matthews, 2016), President Trump appears far more likely than former President Barack Obama to preemptively attack a small rival state like North Korea or even a rival great power like China. In January 2017, the Bulletin of the Atomic Scientists set its Doomsday Clock the closest it has been to midnight since 1953, arguing that “the president’s intemperate statements… have already made a bad international security situation worse” (Hennigan and Wilkinson, 2017). Should they similarly perceive an intemperate and “zero-sum Trump,” Chinese, North Korean, and other foreign leaders may themselves become more inclined toward preemptive strikes. In a nuclear world, the lunacy of even “defensive” preemptive aggression makes a better understanding of its causes imperative.

### Limitations and Future Directions

Our participants were university students (study 1) and ordinary adults (study 2). It is likely that the psychological mechanisms that we uncovered also apply to the political elites who make foreign policy decisions and the military officers who make local combat decisions. They are, after all, fellow citizens socialized within the same national polities. However, there are also other psychological triggers that may shape the preemptive strike decisions that politicians and military personnel make. For instance, politicians may be more driven by partisanship than ordinary citizens. Similarly, honor and reputation may shape the decisions of military personnel more than ordinary citizens.

Another limitation is that the current study investigates preemptive defensive aggression in a simple two-person situation. Interstate interactions are complex processes influenced by both macro-level (structural) and micro-level (psychological) factors. Moreover, decision-making in groups has psychological characteristics (e.g., ambiguous responsibility) that cannot be reduced to individual decision-making. Previous research has indicated that collective group decision making induces greater competition than individual decisions (for a meta-analytic review, see Wildschut et al., 2003). It is thus likely that decisions to engage in preemptive strikes made in group contexts involve different dynamics than does preemptive aggression between individual members of different groups.

We explored the expected rate of outgroup aggression as a proximal driver of preemptive strike rates. However, these expectations did not fully account for the occurrence of preemptive aggression. Across both studies, participants’ preemptive strike rates were often *less frequent* than expected rates of outgroup aggression. It is thus likely that pro-social motivations, such as fairness (Fehr and Fischbacher, 2002) and morality (Schlösser et al., 2015), reduced aggression. On the other hand, in some contexts (e.g., in Study 1’s Japan-China PSG) participants struck more frequently than their expected rates of outgroup attacks. The specific intergroup context may make people overreact to potential threat, or use it as an excuse to engage in what is actually offensive aggression. Future studies should continue exploring dispositional and situational factors that motivate or mitigate preemptive strikes.

Study 1 revealed cross-cultural differences in the drivers of preemptive strikes (e.g., the facets of perceived bilateral relations and the dimensions of social stereotypes). Ample evidence has shown that cultural contexts, such as values and norms (e.g., Gelfand and Realo, 1999; Jing and Bond, 2015), moderate human cooperation and competition. Future research should unpack cultural differences more systematically to better understand preemptive aggression in IR.

## Author Note

Parts of an earlier version of this work were presented at the 23rd International Congress of the International Association for Cross-Cultural Psychology in Nagoya, Japan (July, 2016), and at the 2016 Annual Meeting of the International Society of Political Psychology in Warsaw, Poland (July, 2016).

## Ethics Statement

This study was carried out in accordance with the recommendations of APA Ethical Guidelines for Human Research with written informed consent from all subjects. All subjects gave written informed consent in accordance with the Declaration of Helsinki. The protocol was approved by the University of Delaware IRB, the University of Oklahoma IRB, and Hokkaido University ethics committee.

## Author Contributions

YJ and PG developed the study concept. All authors contributed to the study design. Testing and data collection were performed by YL, AS, LB, and YJ. YJ performed the data analysis and interpretation under the supervision of PG. YJ and PG drafted the manuscript, and AS provided critical revisions. All authors approved the final version of the manuscript for submission.

## Conflict of Interest Statement

The authors declare that the research was conducted in the absence of any commercial or financial relationships that could be construed as a potential conflict of interest.

## Footnotes

1 Currency exchange rates were based on the Big Mac index (The Economist, 2015), correcting for purchasing power parity.

2 For all repeated measures ANOVAs, Greenhouse-Geisser correction was used when the assumption of sphericity was not met.

3 In all multiple comparisons, *p*-values were adjusted based on Bonferroni correction.

4 This is a quadratic trend effect, indicating that the expected attacking rates for Americans and for other Japanese were similar.

5 Additional analyses found that, across all three PSG decisions (intergroup and within-nation), American preemptive strikes were based more on perceived competence of the other participant’s national groups, whereas both Chinese and Japanese preemptive strikes were based more on perceived warmth of the other participant’s national groups. This suggests some cross-cultural differences. Please see the Supplemental Materials.

6 It has not been suggested that stake size matters to PSG decisions. United States–United States attacking rates in Study 1 and Study 2 are similar, also suggesting limited influence of stake size on PSG decisions.

7 The frequencies of preemptively striking against other Americans were also lower under the condition of more positive media framing. But this difference could not be attributed to our experimental manipulation, because participants completed United States–United States PSG before watching the video clip. The different initial attacking proclivities (as manifested in the United States–United States practice PSG) between the two experimental conditions further justified the need for controlling for individual attacking proclivities in our analysis (a repeated measures regression across practice and intergroup PSGs).
