# Supplementary material for: War or Peace? How the Subjective Perception of Great Power Interdependence Shapes Preemptive Defensive Aggression
Source: Front Psychol. 2017 Jun 2;8:864. doi: 10.3389/fpsyg.2017.00864 (PMC5455139; doi:10.3389/fpsyg.2017.00864)
Supplement: Supplementary file 1 [file Presentation_1.pdf]

Table of Contents

Study 1 2

Instructions and Stimuli of the PSG Experiment 2

English Materials 2

Chinese Materials. 8

Japanese Materials. 14

Other Measures in the Pre-PSG Survey 22

Other Measures in the Post-PSG Survey 22

Zero-Order Correlations between Perceived Bilateral Relations and National Stereotypes 23

Additional Analyses of How Preexisting National Stereotypes Shape Preemptive Strikes 25

Study 2 27

Other Survey Measures in the Online Experiment 27

Perceptions of the Interviewee and the News Clip 27

Anger Mediates the Effect of Media Manipulation on Preemptive Strikes 28

Replication Data 31

Study 1

Instructions and Stimuli of the PSG Experiment

Below, we provide screenshots of instructions and stimuli of the red-button PSG, as well as the red-blue-button PSG, in three different languages. This experiment was implemented using the Qualtrics online survey engine.

English Materials.

The Red-Button PSG

Page 1

A three round international decision task

In this decision task, you will participate in THREE rounds of decision making.

In each round, you will play with DIFFERENT participants from three DIFFERENT countries: the U.S., China, and Japan.

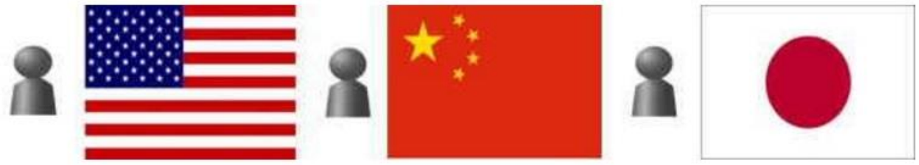

All participants in this decision task are ANONYMOUS, EXCEPT for your NATIONALITY.

We will tell you where the other participant comes from, and the other participant will know that you are from the U.S. But **we WILL NOT disclose any other personal information about either of you**.

Page 2

Payoffs

We will pay all participants CASH based on their performance.

Participants may earn **$14**, **$12**, or nothing (**$0**) from a single round of the decision task, based on their combined decisions.

We will randomly select ONE of the three rounds which you participate in, and pay you based upon your performance in that round.

You should therefore **take EACH round seriously**.

Page 3

Due to the time differences between the U.S., China, and Japan, in each round you and the other participant will make your decisions separately.

After data collection, we will RANDOMLY select one decision made by one participant from each of the US, China, or Japan to pair with your three decisions. Your pay-off will be generated by this random pairing process.

Your decision feedback and cash payment, if any, will first be sealed in a small envelope only using your random ID number. Someone else will then put it in the larger envelope you already put your name on for you to pick up later.

This process ensures that your decisions and cash payoff will remain completely ANONYMOUS to everyone involved.

Page 4

Summary of the task, I

Basic rules: In each round of decision-making, you and another participant will have 30 seconds to decide whether or not to click a RED button on the screen.

Possible outcomes:
1) If NEITHER of you click the button within 30 seconds, BOTH you and the other participant will walk away with the maximum **$14** reward.

2) If one participant clicks the button BEFORE the other participant does, the person who FIRST clicks will only lose **$2**, walking away with **$12**. But the OTHER participant will lose ALL **$14**, walking away with NOTHING (**$0**). Clicking second has no effect, so you cannot retaliate.

Page 5

Summary of the task, II

International decision making: You will play three rounds with DIFFERENT participants from three DIFFERENT countries: the U.S., China, and Japan. All participants in this decision task are ANONYMOUS, EXCEPT for your NATIONALITIES.

Payments: Your three decisions will be randomly paired with three other participants' decisions when all data collection is complete. One of the three rounds will then be randomly selected and you may be paid by cash based on your and the other participant's decisions in the round.

Please raise your hand if you have any questions. Otherwise please proceed to the three round international decision task.

Page 6 (instruction for the US-US PSG)

Round One

The OTHER participant in this first round is an AMERICAN. He or she knows that YOU are AMERICAN too.

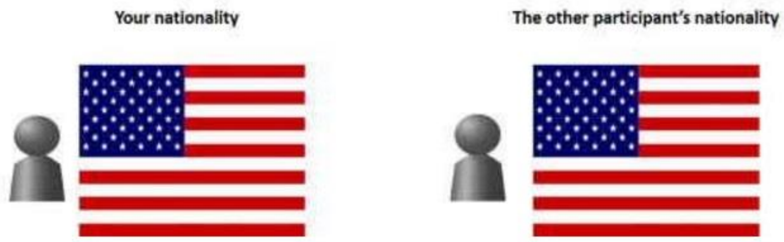

If you do click the red button, **please only do so once.** And please DO NOT click anywhere else on the page.

Once you are ready for the decision task, please proceed to the next page.

Page 7 (5-second count down for the US-US PSG)

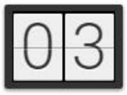

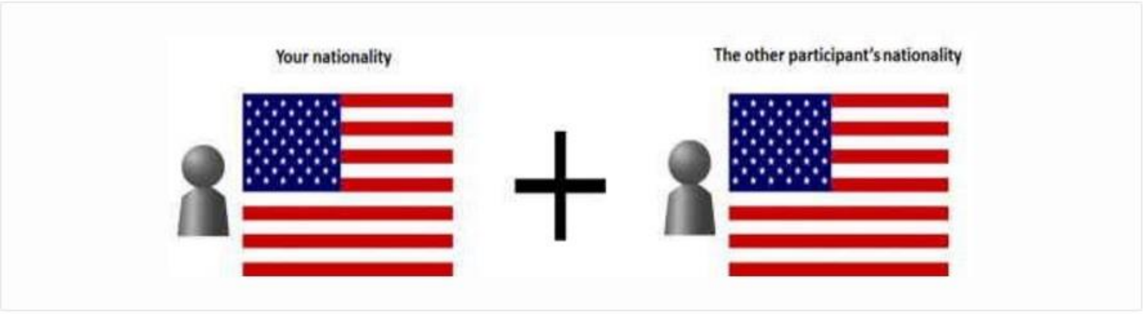

Page 8 (30-second decision period for the US-US PSG)

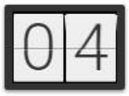

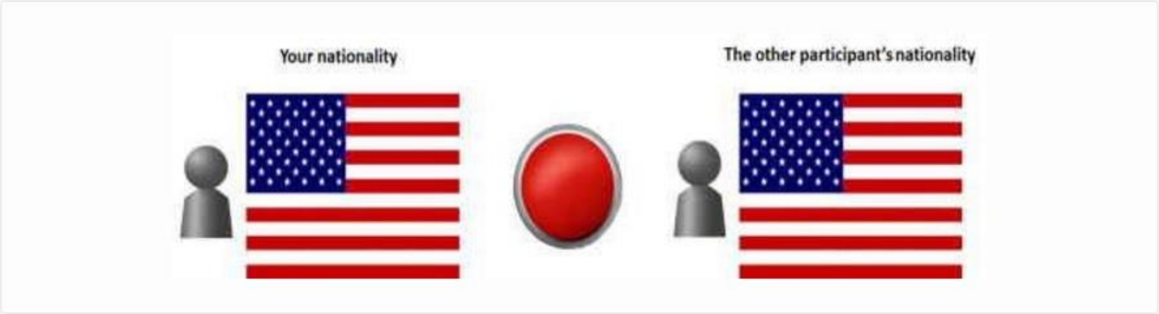

The Red-Blue-Button PSG

Page 1

Additional decision task: The BLUE OR RED button task

You have finished all three rounds and clicked the RED button at least once.

Now, you have the option to **switch your decision(s) to click the RED button by clicking a BLUE button instead**. But changing your decision may have monetary consequences.

Clicking the BLUE button will again cost you **$2 (you will earn $12)**, but unlike clicking the RED button it will not cause the other participant to lose everything; **the other participant will ALSO receive $12**.

The same applies if the OTHER participant clicked the RED button before you did in a previous round. **If he or she clicks the BLUE button instead**, you will receive **$12** instead of **$0** like before.

In any case, the decision made by the participant who clicked the RED button FIRST in the earlier round will determine the payoff outcome for that round.

Please proceed to the next page and decide whether or not to change your decision(s). There will be no timer. If you have any questions, please raise your hand.

Page 2 (for the US-US PSG)

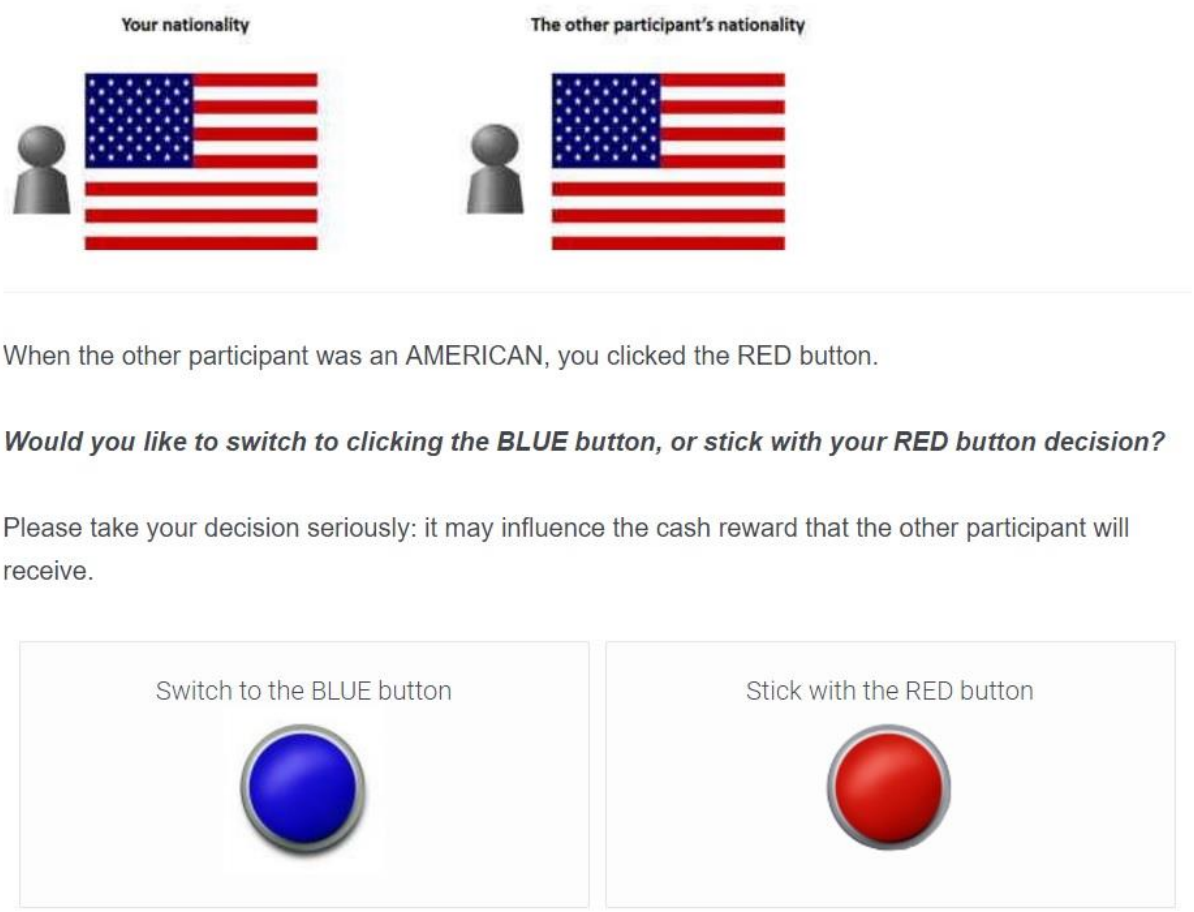

Chinese Materials.

The Red-Button PSG

Page 1

三回合的跨国决策任务

在本决策任务中，您将参与三回合的决策。

每一回合，您将面对不同的参与者，他们分别来自不同国家（共三国）。这些国家包括：中国，美国以及日本。

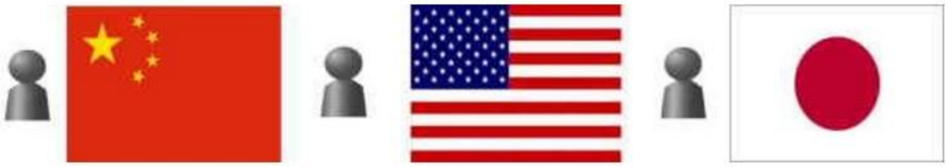

所有参与者在本任务中都是匿名的，但您们的国籍信息除外。

我们将告诉您另一参与者来自哪个国家。另一参与者也会知道您来自中国。但我们**不会透露其它任何有关您们个人身份的信息**。

Page 2

报酬

我们将根据参与者的决策给予现金报酬。

基于您和对方不同决策的组合，参与者可能会从某一回合中挣得**46**元，**41**元或者一无所获（**0**元）。

我们将从您所参与的三回合决策中随机抽取其中之一，并根据您在该回合的决策支付报酬。

因此您应当**认真的对待每一回合**。

Page 3

由于中、美、日三国存在时差，每一回合您和另一参与者将单独进行决策。

收集完数据后，我们将在中、美、日三个国家各随机选出一名参与者的决策与您在相应回合中的决策配对。这一随机配对的结果将决定您的报酬。

随机配对后，我们会首先派人仅仅根据您的随机实验编号，将您的决策结果反馈以及现金报酬（如果有的话）密封于一个小信封内。接下来，我们将另派他人将密封了的小信封放入您已经写下自己学号和名字的那个信封内，供您之后前来领取。

这一程序将保证您的决策结果对任何参与本研究的人员都是完全保密的。

Page 4

任务总结（一）

基本规则: 每一回合的决策，您与另一参与者将有30秒的时间决定是否点击屏幕上的红色按钮。

可能的结果:
1) 如果30秒内你们都没有点击按钮，您与另一参与者都将获得**46**元的最高现金奖励。

2) 如果某一参与者在另一参与者之前点击按钮，首先点击按钮的人将仅损失**5**元、拿到**41**元。但另一参与者将损失所有的**46**元，而一无所获（**0**元）。一旦有人首先点击了，之后的一切点击都将失去效果，因此您无法进行报复。

Page 5

任务总结（二）

跨国决策: 您将进行三回合的决策；每回合的另一参与者是不同的人，分别来自三个不同的国家：中国，美国，日本。本决策任务的所有参与者都是匿名的，除了你们的国籍信息。

报酬: 所有数据收集完毕后，您的三次决策将与另外三位参与者的决策随机配对。我们将随机选出三回合的其中之一；基于您以及另一参与者在该回合的决策，您可能会获得现金奖励。

如果您有任何问题，请举手示意。否则，请前进至下一页面完成三回合的跨国决策任务。

Page 6 (instruction for the China-US PSG)

第一回合

本回合的另一参与者是美国人。他或者她知道您是中国人。

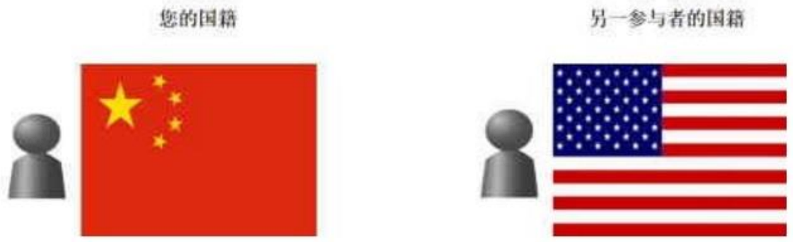

如果您的确要点击红色按钮，**请只点击一次**。另外请不要点击页面的其他部分。

一旦您准备好了进行决策任务，请前进至下一页面。

Page 7 (5-second count down for the China-US PSG)

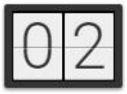

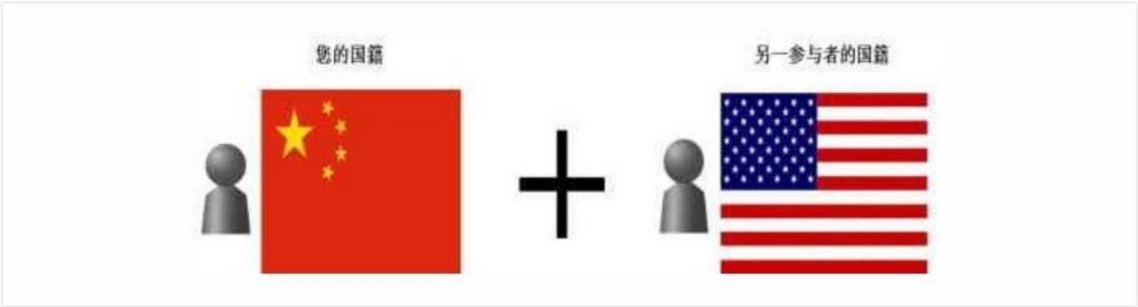

Page 8 (30-second decision period for the China-US PSG)

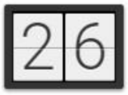

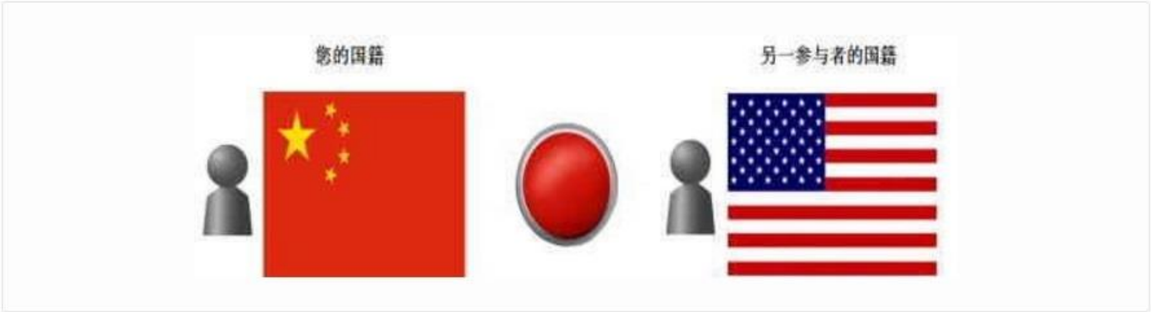

The Red-Blue-Button PSG

Page 1

附加决策任务：蓝色或红色按钮任务

您已经完成了所有三回合的决策，并至少点击了红色按钮一次。

现在，您有机会**改变之前点击红色按钮的决定，变成点击一个蓝色的按钮**。但改变先前的决定可能会影响现金收益的结果。

点击蓝色按钮也将令您损失**5元**（**您将获得41元**）。但与点击红色按钮不同，点击蓝色按钮将不会导致另一参与者损失一切；**另一参与者在此条件下也将获得41元。**

如果之前的某一回合另一参与者比您更早地点击了红色按钮，上述规则同样适用。**如果他或者她转而点击蓝色按钮**，您将得到**41**元而不是之前的**0**元。

总之，之前回合中谁首先点击红色按钮，则他或者她的决策将决定该回合最终的收益结果。

请前进至下一页面决定是否改变您的决策。我们将不再进行倒计时。如果您有任何问题，请举手示意。

Page 2 (for the China-US PSG)

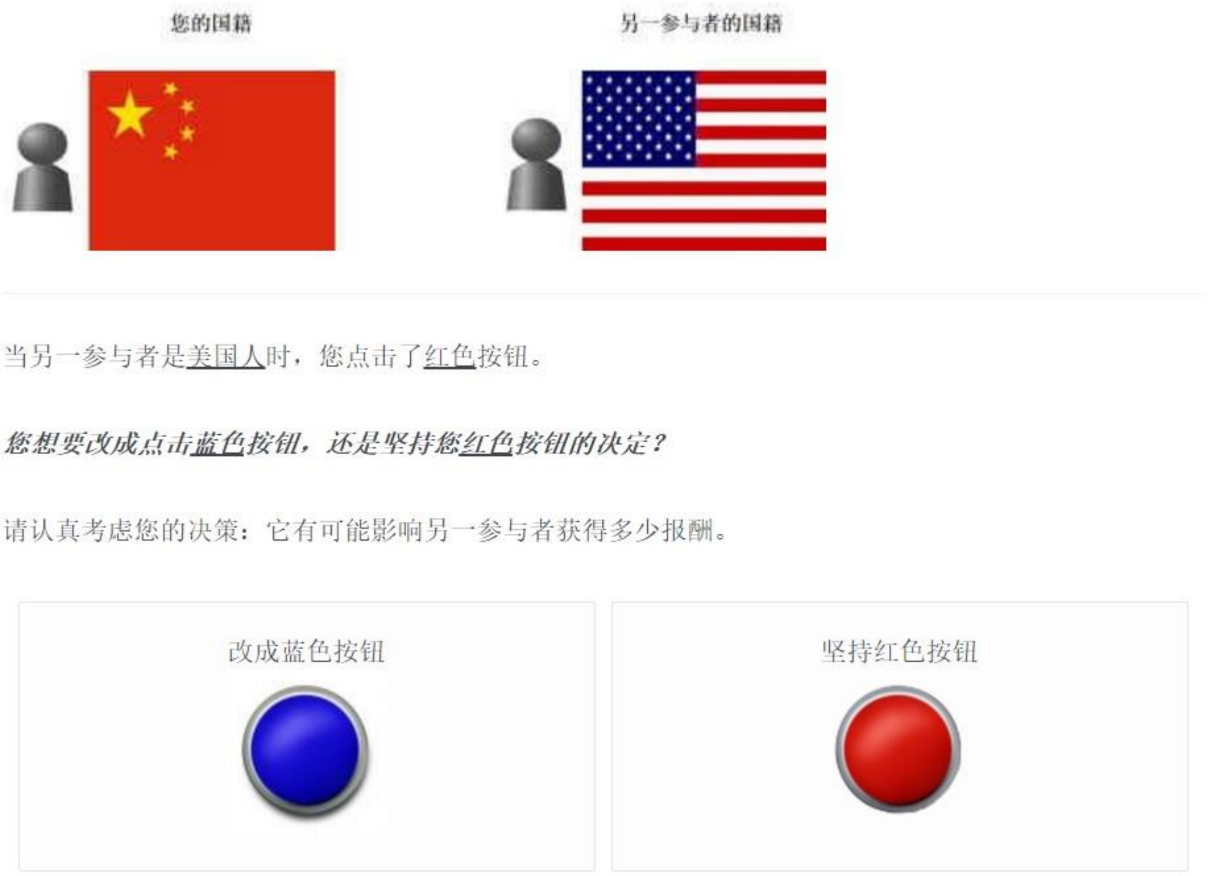

Japanese Materials.

The Red-Button PSG

Page 1

相手の国籍について

この実験では、先ほど説明した決定作業を、**３回**行っていただきます。

３回それぞれでは、あなたは別々の３人の相手と組み合わさって、決定します。

組み合わさる相手はそれぞれ日本人、アメリカ人、中国人です。あなたの相手になる人は、それぞれの国の実験室で実験に参加しています。

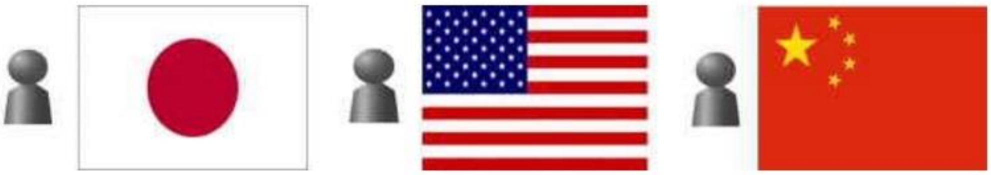

あなたも含め、実験に参加している参加者の決定はすべて匿名で行われます。 ただし、それぞれの**国籍の情報だけ**が、相手に分かります。

Page 2

実験の報酬について

実験参加の報酬は、決定の結果に応じて、**現金**で支払われます。

あなたと相手の決定によって、あなたがもらえるお金は１０００円、９００円、もしくは０円のいずれか１つになります。
あなたは３人の相手と合計３回の決定を行いますが、その中から**ランダムに１回**が選ばれます。
選ばれた１回の中であなたが得たお金が、この実験におけるあなたの報酬になります。
そのため、**すべての回の決定を真剣に行ってください。**

Page 3

この実験は実際に日本、アメリカ、中国の実験室で行われています。

ただし、時差の問題により、それぞれの国の参加者がリアルタイムに同時参加することが出来ません。 そのため決定の結果は、以下のような方法で決まります。

それぞれの参加者はまず、相手がいる時と同じように決定を行います。
次に、すべての参加者の決定のデータがそろった後、**参加者のデータは実際に組み合わされ、結果に反映されます。** 誰と組み合わさるか、どの回の決定が実際の参加報酬に反映されるかは、**ランダム**に決まります。

あなたに支払う報酬は、あなたと対面しない、かつ実験参加ID以外の情報について知らない、別の担当者が集計します。
こうすることによって、この実験であなた個人の決定の匿名性が守られます。

Page 4

説明のまとめ１

意思決定の内容： あなたはもう一人の参加者と二人一組で意思決定を行います。 画面に現れる赤いボタンを３０秒以内に押すかどうかを決めてください。

結果について：
① あなたも相手も３０秒間ボタンを押さなければ、あなたも相手も**元手の１０００円**をそのままもらいます。

② どちらか一方が先にボタンを押した場合、**先に押したほうは１００円を失い、９００円をもらいます。**しかし相手に先に**押されたほうは１０００円を全部失い、もらえるお金が０円になります。**
後からボタンを押しても結果は変わりません。

Page 5

説明のまとめ２

国際実験：あなたは**３つの国の相手とそれぞれ１回ずつ意思決定**を行います。相手は日本人、中国人、アメリカ人参加者です。決定はすべて**匿名**に行われますが、相手にはあなたの**国籍だけが知らされます。**

報酬の支払いについて：３つの国の相手それぞれと決定を行ったあと、あなたの決定はこれらの国の参加者の決定と**ランダム**に組み合わされます。３つの結果のうち**１つ**がランダムに選ばれ、あなたはその結果通りの報酬を現金で受け取ることができます。

質問がある方は遠慮なく実験担当に質問してください。質問がない方は、次に進んでください。

次のページから、実際の決定が始まります。

Page 6 (instruction for the Japan-US PSG)

１回目の決定

この回の相手は**アメリカ人**です。

相手はあなたが日本人であることを知っています。

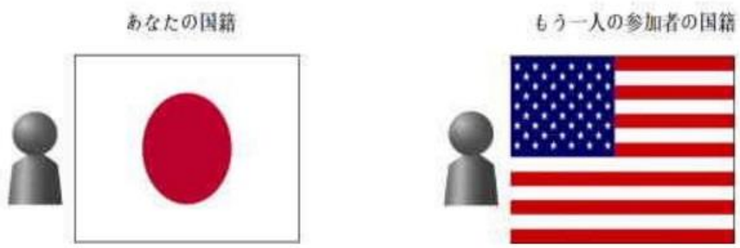

赤いボタンを押す場合には、**１回だけ**押してください。また、ボタン以外の部分を押さないように注意してください。

準備ができたら次のページに進んでください。５秒のカウントダウンが始まります。

Page 7 (5-second count down for the Japan-US PSG)

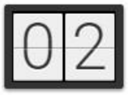

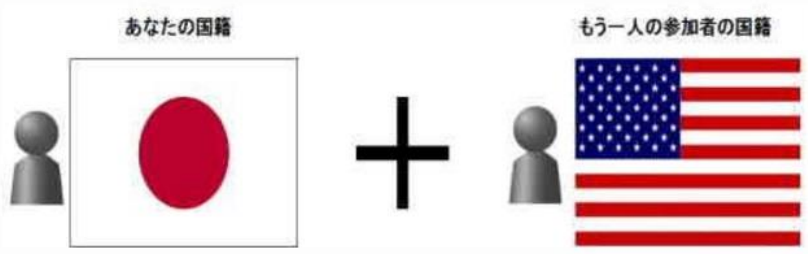

Page 8 (30-second decision period for the Japan-US PSG)

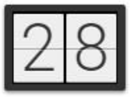

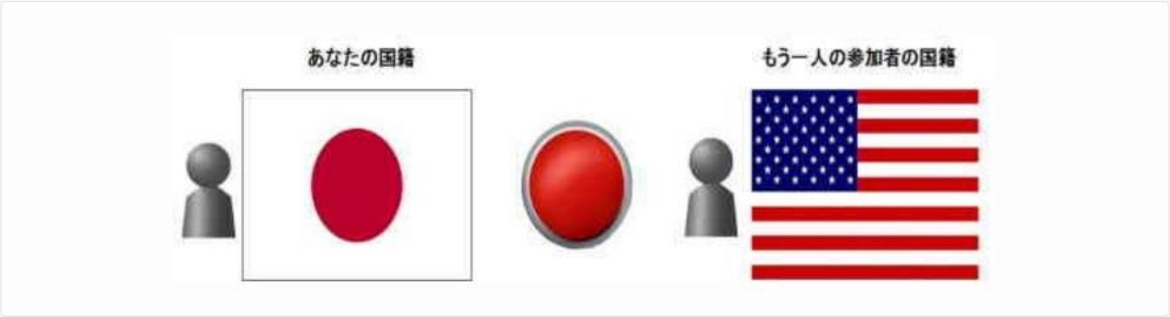

The Red-Blue-Button PSG

Page 1

追加意思決定：青いボタンと赤いボタンの選択

あなたは３回の決定すべてを行いました。また、決定中には少なくとも１回赤いボタンを押しました。

ここで、先ほどの決定を変えるチャンスがあります。

あなたが押したボタンを、赤いボタンの代わりに青いボタンに切り替えるチャンスがあります。

青いボタンに切り替えた場合にも、あなたが**ボタンを押すために支払った１００円はそのまま支払われます。**（つまり青いボタンに切り替えた場合でも、あなたがもらうお金は９００円に変わりありません）

しかし、赤いボタンと青いボタンのどちらを選ぶかによって**相手がもらう金額が変わります。**

赤いボタンを押した場合、相手は元手をすべて失い、もらえるお金がゼロになります。 しかし、**あなたが青いボタンに切り替えると、相手も９００円をもらえます。**

同様に、もし先ほどの決定の中で相手が先に赤いボタンを押していたとしたら、相手が青いボタンに切り替えれば、あなたはゼロ円ではなく**９００円をもらう**ことになります。
いずれの場合でも、**先にボタンを押した人**がどのボタンを選ぶかによって、実際の結果が決まります。

以上の説明をよく読み、赤いボタンのままにするか、それとも青いボタンに切り替えるかを決定してください。 この決定は実際にあなたと相手の報酬に反映されますので、よく考えてください。

準備ができましたら、次のページに進んでください。

Page 2 (for the Japan-US PSG)

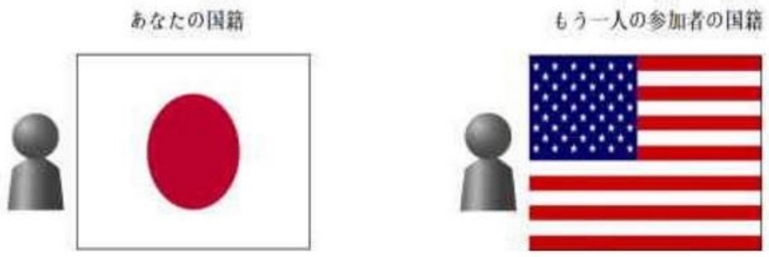

先ほど**アメリカ人**の相手と行った決定の中で、あなたは赤いボタンを押しました。

あなたはアメリカ人相手に対する決定を青いボタンに切り替えますか？それとも赤いボタンのままにしますか？

この決定は実際にあなたと相手の報酬に影響しますので、よく考えて決定してください。

青いボタンに切り替える

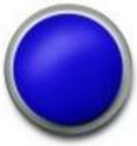

赤いボタンのままにする

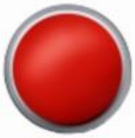

Other Measures in the Pre-PSG Survey

In addition to preexisting perceptions of bilateral relations and national stereotypes, we also implemented the following measures.

Personality-Like Individual Differences. These measures include social value orientations, endorsements of personal values, generalized trust, social risk-taking tendencies, aggressive personality, patriotism, group entitativity, and culture of honor.

Historical Beliefs and Media Exposure. Participants were asked various questions regarding their historical beliefs of current interest (e.g., Americans' views on the Pacific war and the Korean war, Chinese and Japanese views on the Sino-Japan war in WWII), as well as their media exposure to realistic conflict between countries of interest (e.g., US-China trade disputes, China-Japan territorial disputes).

Demographic Variables. At the end of the survey, participants reported their genders, ages, home states/provinces/counties, political affiliations or/and political positions (liberal-conservative for American participants; left-right for Chinese and Japanese participants).

Other Measures in the Post-PSG Survey

In addition to the expected rates of outgroup preemptive strikes and hypothetical unilateral PSG, we also implemented the following measures.

Understanding of the PSG Rules. Following the PSG decision task, participants answered four multiple-choice questions assessing their understandings of the PSG pay-off rules. Participants who failed to recall the correct rules for the red-button PSG were excluded from all analyses (US: N = 6; China: N =15; Japan: N = 3), whereas participants who failed to recall the correct rules for the red-blue-button PSG (US: N = 24; China: N = 26, Japan: N = 5) were only excluded from the

analysis of the red-blue-button decisions. Our major findings remained robust with or without excluding participants who had not understood the task.

Other Measures. Participants also were asked a number of questions regarding their other possible motives for the PSG decision (e.g., maximizing one's self-interest, mistrust, interpersonal competition, etc.), their anxiety of making the red-button decision, and their goals in the PSG task (e.g., cooperation, manage anxiety, etc.). Additionally, each participant made decision in a hypothetical stag hunt game facing a different person from each of the three countries; this is a measure tapping into trust and cooperation.

Zero-Order Correlations between Perceived Bilateral Relations and National Stereotypes

Table S1 to S3 below displays correlations between perceptions of bilateral relations and stereotypes against foreigners, for each of the three samples separately.

Table S1

Zero-Order Correlations for the American Sample

Correlations

		US-China Competition	US-Japan Competition	Likelihood of US-China Military Conflict	Likelihood of US-Japan Military Conflict	Optimism about Future US-China Relations	Optimism about Future US-Japan Relations	Chinese Warmth	Japanese Warmth	Chinese Competence	Japanese Competence
US-China Competition	Pearson Correlation	1	.359**	.215*	-.065	-.348**	-.059	-.204*	-.040	.016	.124
	Sig. (2-tailed)		.000	.021	.492	.000	.530	.029	.675	.866	.188
	N	115	115	115	115	115	115	115	115	115	115
US-Japan Competition	Pearson Correlation	.359**	1	.074	.393**	-.038	-.473**	-.301**	-.388**	.052	-.038
	Sig. (2-tailed)	.000		.432	.000	.688	.000	.001	.000	.584	.686
	N	115	115	115	115	115	115	115	115	115	115
Likelihood of US-China Military Conflict	Pearson Correlation	.215*	.074	1	.484**	-.363**	-.172	-.244**	-.164	-.136	-.026
	Sig. (2-tailed)	.021	.432		.000	.000	.065	.009	.079	.146	.780
	N	115	115	115	115	115	115	115	115	115	115
Likelihood of US-Japan Military Conflict	Pearson Correlation	-.065	.393**	.484**	1	-.171	-.546**	-.328**	-.344**	-.029	-.236*
	Sig. (2-tailed)	.492	.000	.000		.067	.000	.000	.000	.758	.011
	N	115	115	115	115	115	115	115	115	115	115
Optimism about Future US-China Relations	Pearson Correlation	-.348**	-.038	-.363**	-.171	1	.487**	.216*	.007	.164	.017
	Sig. (2-tailed)	.000	.688	.000	.067		.000	.020	.941	.080	.853
	N	115	115	115	115	115	115	115	115	115	115
Optimism about Future US-Japan Relations	Pearson Correlation	-.059	-.473**	-.172	-.546**	.487**	1	.299**	.294**	.136	.302**
	Sig. (2-tailed)	.530	.000	.065	.000	.000		.001	.001	.147	.001
	N	115	115	115	115	115	115	115	115	115	115
Chinese Warmth	Pearson Correlation	-.204*	-.301**	-.244**	-.328**	.216*	.299**	1	.676**	.243**	.353**
	Sig. (2-tailed)	.029	.001	.009	.000	.020	.001		.000	.009	.000
	N	115	115	115	115	115	115	115	115	115	115
Japanese Warmth	Pearson Correlation	-.040	-.388**	-.164	-.344**	.007	.294**	.676**	1	.228*	.495**
	Sig. (2-tailed)	.675	.000	.079	.000	.941	.001	.000		.014	.000
	N	115	115	115	115	115	115	115	115	115	115
Chinese Competence	Pearson Correlation	.016	.052	-.136	-.029	.164	.136	.243**	.228*	1	.624**
	Sig. (2-tailed)	.866	.584	.146	.758	.080	.147	.009	.014		.000
	N	115	115	115	115	115	115	115	115	115	115
Japanese Competence	Pearson Correlation	.124	-.038	-.026	-.236*	.017	.302**	.353**	.495**	.624**	1
	Sig. (2-tailed)	.188	.686	.780	.011	.853	.001	.000	.000	.000	
	N	115	115	115	115	115	115	115	115	115	115

**. Correlation is significant at the 0.01 level (2-tailed).

*. Correlation is significant at the 0.05 level (2-tailed).

Table S2

Zero-Order Correlations for the Chinese Sample

Correlations

		China-US Competition	China-Japan Competition	Likelihood of China-US Military Conflict	Likelihood of China-Japan Military Conflict	Optimism about Future China-US Relations	Optimism about Future China-Japan Relations	American Warmth	Japanese Warmth	American Competence	Japanese Competence
China-US Competition	Pearson Correlation	1	.445**	.048	.049	-.246*	-.131	-.151	-.018	.063	-.024
	Sig. (2-tailed)		.000	.622	.621	.011	.181	.121	.857	.522	.810
	N	106	106	106	106	106	106	106	106	106	106
China-Japan Competition	Pearson Correlation	.445**	1	.155	.407**	-.124	-.400**	-.108	-.216*	-.130	-.127
	Sig. (2-tailed)	.000		.113	.000	.205	.000	.269	.026	.186	.193
	N	106	106	106	106	106	106	106	106	106	106
Likelihood of China-US Military Conflict	Pearson Correlation	.048	.155	1	.624**	-.280**	-.252**	-.048	-.243*	-.117	-.133
	Sig. (2-tailed)	.622	.113		.000	.004	.009	.628	.012	.231	.175
	N	106	106	106	106	106	106	106	106	106	106
Likelihood of China-Japan Military Conflict	Pearson Correlation	.049	.407**	.624**	1	-.142	-.442**	-.039	-.315**	-.033	-.061
	Sig. (2-tailed)	.621	.000	.000		.147	.000	.689	.001	.736	.533
	N	106	106	106	106	106	106	106	106	106	106
Optimism about Future China-US Relations	Pearson Correlation	-.246*	-.124	-.280**	-.142	1	.508**	.120	-.019	.030	-.025
	Sig. (2-tailed)	.011	.205	.004	.147		.000	.222	.843	.762	.798
	N	106	106	106	106	106	106	106	106	106	106
Optimism about Future China-Japan Relations	Pearson Correlation	-.131	-.400**	-.252**	-.442**	.508**	1	.191*	.254**	.020	-.040
	Sig. (2-tailed)	.181	.000	.009	.000	.000		.050	.009	.836	.681
	N	106	106	106	106	106	106	106	106	106	106
American Warmth	Pearson Correlation	-.151	-.108	-.048	-.039	.120	.191*	1	.537**	.583**	.571**
	Sig. (2-tailed)	.121	.269	.628	.689	.222	.050		.000	.000	.000
	N	106	106	106	106	106	106	106	106	106	106
Japanese Warmth	Pearson Correlation	-.018	-.216*	-.243*	-.315**	-.019	.254**	.537**	1	.393**	.509**
	Sig. (2-tailed)	.857	.026	.012	.001	.843	.009	.000		.000	.000
	N	106	106	106	106	106	106	106	106	106	106
American Competence	Pearson Correlation	.063	-.130	-.117	-.033	.030	.020	.583**	.393**	1	.738**
	Sig. (2-tailed)	.522	.186	.231	.736	.762	.836	.000	.000		.000
	N	106	106	106	106	106	106	106	106	106	106
Japanese Competence	Pearson Correlation	-.024	-.127	-.133	-.061	-.025	-.040	.571**	.509**	.738**	1
	Sig. (2-tailed)	.810	.193	.175	.533	.798	.681	.000	.000	.000	
	N	106	106	106	106	106	106	106	106	106	106

**. Correlation is significant at the 0.01 level (2-tailed).

*. Correlation is significant at the 0.05 level (2-tailed).

Table S3

Zero-Order Correlations for the Japanese Sample

Correlations

		Japan-China Competition	Japan-US Competition	Likelihood of Japan-China Military Conflict	Likelihood of Japan-US Military Conflict	Optimism about Future Japan-China Relations	Optimism about Future Japan-US Relations	Chinese Warmth	American Warmth	Chinese Competence	American Competence
Japan-China Competition	Pearson Correlation	1	.202*	.288**	-.146	-.313**	.109	-.396**	-.012	.006	.004
	Sig. (2-tailed)		.045	.004	.149	.002	.284	.000	.905	.955	.968
	N	99	99	99	99	99	99	99	99	99	99
Japan-US Competition	Pearson Correlation	.202*	1	.071	.109	.111	-.267**	-.146	-.152	-.279**	-.179
	Sig. (2-tailed)	.045		.484	.281	.275	.007	.151	.134	.005	.076
	N	99	99	99	99	99	99	99	99	99	99
Likelihood of Japan-China Military Conflict	Pearson Correlation	.288**	.071	1	.457**	-.417**	-.040	-.331**	-.048	-.042	.095
	Sig. (2-tailed)	.004	.484		.000	.000	.695	.001	.640	.683	.349
	N	99	99	99	99	99	99	99	99	99	99
Likelihood of Japan-US Military Conflict	Pearson Correlation	-.146	.109	.457**	1	-.137	-.247*	-.140	-.032	-.181	-.054
	Sig. (2-tailed)	.149	.281	.000		.175	.014	.167	.757	.073	.594
	N	99	99	99	99	99	99	99	99	99	99
Optimism about Future Japan-China Relations	Pearson Correlation	-.313**	.111	-.417**	-.137	1	.212*	.433**	.107	.175	.075
	Sig. (2-tailed)	.002	.275	.000	.175		.035	.000	.292	.084	.460
	N	99	99	99	99	99	99	99	99	99	99
Optimism about Future Japan-US Relations	Pearson Correlation	.109	-.267**	-.040	-.247*	.212*	1	.049	.174	.173	.299**
	Sig. (2-tailed)	.284	.007	.695	.014	.035		.628	.086	.086	.003
	N	99	99	99	99	99	99	99	99	99	99
Chinese Warmth	Pearson Correlation	-.396**	-.146	-.331**	-.140	.433**	.049	1	.090	.335**	.078
	Sig. (2-tailed)	.000	.151	.001	.167	.000	.628		.373	.001	.443
	N	99	99	99	99	99	99	99	99	99	99
American Warmth	Pearson Correlation	-.012	-.152	-.048	-.032	.107	.174	.090	1	.038	.283**
	Sig. (2-tailed)	.905	.134	.640	.757	.292	.086	.373		.712	.004
	N	99	99	99	99	99	99	99	99	99	99
Chinese Competence	Pearson Correlation	.006	-.279**	-.042	-.181	.175	.173	.335**	.038	1	.404**
	Sig. (2-tailed)	.955	.005	.683	.073	.084	.086	.001	.712		.000
	N	99	99	99	99	99	99	99	99	99	99
American Competence	Pearson Correlation	.004	-.179	.095	-.054	.075	.299**	.078	.283**	.404**	1
	Sig. (2-tailed)	.968	.076	.349	.594	.460	.003	.443	.004	.000	
	N	99	99	99	99	99	99	99	99	99	99

*. Correlation is significant at the 0.05 level (2-tailed).

**. Correlation is significant at the 0.01 level (2-tailed).

Additional Analyses of How Preexisting National Stereotypes Shape Preemptive Strikes

Unlike the analysis performed in the text (stereotypes against two groups of foreigners), here we included reported national stereotypes about *all* three groups of people including one's compatriots.

Using the GEE logistic regression (GEE), the decision to click the red button (across all three conditions) was regressed onto perceived warmth and competence of people in each of the three countries, for the three samples separately.

American Participants. Regressing the red-button decision onto the two stereotype components simultaneously did not yield any significant, unique predictor (warmth: $p > .250$; competence: $p = .120$). However, perceived competence *alone* was a significant predictor of American participants' PSG attacks, Exp (B) = .75, $p = .043$, 95% CI = [.57, .99]; the more competent the other participant's national group was perceived, the less likely American participants were to

click the red button against that group. By contrast, perceived warmth alone was not a significant predictor, Exp (B) = .80, p = .131, 95% CI = [.61, 1.07]. These main effects were not qualified by the other participant's nationality (ps > .250).

Chinese Participants. Regressing the red-button decision onto the two stereotype components simultaneously did not yield any significant, unique predictor (warmth: p = .074; competence: p > .250). However, perceived warmth *alone* was a significant predictor of Chinese participants' PSG attacks, Exp (B) = .77, p = .019, 95% CI = [.62, .96]; the warmer the other participant's national group was perceived, the less likely Chinese participants were to click the red button against that group. By contrast, perceived competence alone was not a significant predictor, Exp (B) = .81, p = .087, 95% CI = [.64, 1.03]. These main effects were not qualified by the other particpant's nationality (ps > .250).

Japanese Participants. Regressing the red-button decision onto the two stereotype components simultaneously, perceived warmth was the only significant, *unique* predictor of Japanese participants' PSG attacks, Exp (B) = .77, p = .004, 95% CI = [.65, .92]; the warmer the other participant's national group was perceived, the less likely Japanese participants were to click the red button against that group. By contrast, perceived competence was not a significant, unique predictor, Exp (B) = 1.04, p > .250, 95% CI = [.80, 1.36]. These main effects also were not qualified by the other participant's nationality (interaction term for Warmth: p = .192; interaction term for competence: p > .250).

Taken together, these results suggested that American PSG attacks were based more on expectations about of the other party's capabilities for cooperation (competence), whereas Chinese and Japanese PSG attacks were based more on expectations about the other party's intentions for cooperation (warmth). This cross-cultural finding is consistent with previous

research demonstrating that North Americans and East Asians value communion and agency differently in their social judgment (e.g., Chen et al., 2016; Rule et al., 2010).

References

Chen, F. F., Jing, Y., Lee, J. M., & Bai, L. (2016). Culture matters: The looks of a leader are not all the same. *Social Psychological and Personality Science, 7,* 570-578.

Rule, N. O., Ambady, N., Adams, Jr. R. B., Ozono, H., Nakashima, S., Yoshikawa, S., & Watabe, M. (2010). Polling the face: Prediction and consensus across cultures. *Journal of Personality and Social Psychology, 98,* 1-15.

Study 2

Other Survey Measures in the Online Experiment

After CNN media manipulation, we administrated a memory test about the content of the news clip. We measured participants' trust in Chinese, Japanese, Russian, and American people, and their governments. We measured participants' feelings towards China, Japan, Russia, and the US, and their foreign policy preferences (about China, Japan and Russia). After the PSG task, we tested participants' understandings of the PSG rules. Last, they filled out questions regarding their personalities, including nationalism, social dominance, right-wring authoritarianism, uncertainty avoidance, neuroticism, and agreeableness, as well as demographic characteristics.

Perceptions of the Interviewee and the News Clip

Table S4 displays descriptive statistics for perceived personality of person interviewed (likeable, credible, and attractive) and of the news report itself (professional, engaging, balanced, and stimulating), for the "Kiernan" interview (more zero/negative-sum) and the "Paulson" interview (more positive-sum) separately. A MANOVA revealed a huge main effect of media framing on these perceptions, F (7, 119) = 15.08, p <.001, partial η^2 = .47. Specifically, Mr.

Paulson was perceived as more credible and attractive than Mr. Kiernan (*ps* < .01); the "Paulson" interview was also perceived as more professional and balanced than the "Kiernan" interview (*ps* < .001). On the other hand, Mr. Kiernan was perceived as more likeable than Mr. Paulson (*p* = .016). There were no significant differences on other perceptions.

Table S4

Descriptive Statistics for Perceptions of the Interviewee and the News Clip Itself under Different Media Manipulations

Descriptive Statistics

	Condition	Mean	Std. Deviation	N
Likeable	Competitive	3.71	1.575	68
	Cooperative	3.08	1.236	59
	Total	3.42	1.455	127
Credible	Competitive	4.91	1.690	68
	Cooperative	5.73	1.201	59
	Total	5.29	1.533	127
Attractive	Competitive	3.19	1.509	68
	Cooperative	4.75	1.254	59
	Total	3.91	1.594	127
Professional	Competitive	5.01	1.706	68
	Cooperative	6.05	1.195	59
	Total	5.50	1.573	127
Engaging	Competitive	4.82	1.803	68
	Cooperative	5.00	1.543	59
	Total	4.91	1.683	127
Balanced	Competitive	4.06	1.752	68
	Cooperative	5.07	1.350	59
	Total	4.53	1.651	127
Stimulating	Competitive	4.65	1.768	68
	Cooperative	4.58	1.632	59
	Total	4.61	1.700	127

Anger Mediates the Effect of Media Manipulation on Preemptive Strikes

According to Baron and Kenny (1986), there are three requirements to establish a mediational linkage from an independent variable (IV) to an outcome variable (DV) via a

mediator (ME): a) the IV's variability accounts for the DV's variability, b) the IV's variability accounts for the ME's variability, and c) the ME's variability accounts for the DV's variability after controlling for the IV. In particular, the mediational effect via ME should reduce the IV's effect on the DV.

In the text, we have demonstrated that CNN media manipulation shaped both anger about China's rise and preemptive strikes against Chinese people (conditions a and b are satisfied). To demonstrate that condition c can also be met, we conducted a GEE regression in which the two intergroup PSG decisions (against Chinese and Japanese) were regressed onto an interaction between media framing and the other participant's nationality, and an interaction between angry and the other participant's nationality simultaneously (all three variables' main effects were also included); the two interactions assess media framing and anger's effects across the two intergroup preemptive strikes.

The results revealed a significant interaction between angry and the other participant's nationality, $\chi^2 = 4.03$, $df = 1$, $p = .045$. This demonstrates the unique effect of angry on the outcome (condition c is satisfied); simple slope test further indicated that when participants were angrier, they were more likely to attack Chinese than Japanese in the intergroup PSG (1 *SD* more angry above average: $M_{\text{difference}} = 15\%$, $p = .006$, 95% CI = [4%, 26%]; 1 *SD* less angry below average: $M_{\text{difference}} = 2\%$, $p > .250$, 95% CI = [-5%, 8%]). Importantly, the interaction between media framing and the other participant's nationality was substantially reduced after controlling for anger, $\chi^2 = .83$, $df = 1$, $p > .250$ (vs. $\chi^2 = 3.33$, $df = 1$, $p = .068$ before). This suggests that media framing's effect on intergroup preemptive strikes was fully mediated by anger.

Reference

Baron, R. M. & Kenny, D. A. (1986). The moderator–mediator variable distinction in social psychological research: Conceptual, strategic, and statistical considerations. *Journal of Personality and Social Psychology, 51*, 1173-1182.

Replication Data

The datasets analyzed in our studies can be found via the following web links. Study 1 American Data: https://www.dropbox.com/s/mkee04vr2kyrsb3/Study1_US.sav?dl=0 Study 1 Chinese Data: https://www.dropbox.com/s/3sp25o6zmrgwbtl/Study1_China.sav?dl=0 Study 1 Japanese Data: https://www.dropbox.com/s/9kn095j3d5uvnby/Study1_Japan.sav?dl=0 Study 2 MTurk Data: https://www.dropbox.com/s/9e4d98qm0q6q5ax/Study2_MTurk.sav?dl=0 ***Note.*** These datasets are full datasets where no participant has been excluded. To replicate our analyses in the text, please select *only* Study 1 participants who passed our memory test about PSG rules (the variable labeled “Red_Correct” in each Study 1 dataset), and *only* Study 2 participants who *both* passed our memory test about PSG rules (the variable labeled “PSG_Check” in Study 2 dataset) and memory test about CNN interview’s content (the variable labeled “Video_Check” in Study 2 dataset).

SPSS syntax for within-subject PSG data transformation: https://www.dropbox.com/s/slhgglp93mdap3f/Within_Subject_Transform.sps?dl=0 ***Note.*** This transformation is necessary for GEE regression where each participant’s multiple PSG decisions should be recorded in a univariate format.
